# *In Utero* Exposure to Metformin Reduces the Fertility of Male Offspring in Adulthood

**DOI:** 10.3389/fendo.2021.750145

**Published:** 2021-10-18

**Authors:** Mélanie C. Faure, Rita Khoueiry, Jusal Quanico, Hervé Acloque, Marie-Justine Guerquin, Michael J. Bertoldo, Claire Chevaleyre, Christelle Ramé, Isabelle Fournier, Michel Salzet, Joëlle Dupont, Pascal Froment

**Affiliations:** ^1^ l’Institut National de Recherche Pour l’Agriculture, l’Alimentation et l’Environnement (INRAE), UMR85 Physiologie de la Reproduction et des Comportements/Centre national de la Recherche Scientifique (CNRS), UMR7247/Université François Rabelais de Tours/Institut français du Cheval et de l'Équitation (IFCE), Nouzilly, France; ^2^ Epigenetics Group, International Agency for Research on Cancer (IARC), Lyon, France; ^3^ Université Lille 1, INSERM U1192 - Protéomique Réponse Inflammatoire Spectrométrie de Masse (PRISM), Villeneuve d’Ascq, France; ^4^ Université Paris-Saclay, INRAE, AgroParisTech, Génétique Animale et Biologie Intégrative (GABI), Jouy-en-Josas, France; ^5^ UMR967 INSERM, Commissariat à l'Énergie Atomique (CEA)/Direction de la Recherche Fondamentale (DRF)/Institut de Radiobiologie Cellulaire et Moléculaire (iRCM)/Service Cellules Souches et Radiation (SCSR)/LDG, Université Paris Diderot, Sorbonne Paris Cité, Université Paris-Sud, Université Paris-Saclay, Laboratory of Development of the Gonads, Fontenay aux Roses, France; ^6^ Fertility and Research Centre, School of Women’s and Children’s Health, University of New South Wales, Sydney, NSW, Australia; ^7^ School of Medical Sciences, University of New South Wales, Sydney, NSW, Australia

**Keywords:** metformin, embryo, pregnancy, ovary, testis, spermatozoa

## Abstract

Metformin is a drug used for the treatment of type 2 diabetes and disorders associated with insulin resistance. Metformin is also used in the treatment of pregnancy disorders such as gestational diabetes. However, the consequences of foetal exposure to metformin on the fertility of exposed offspring remain poorly documented. In this study, we investigated the effect of *in utero* metformin exposure on the fertility of female and male offspring. We observed that metformin is detectable in the blood of the mother and in amniotic fluid and blood of the umbilical cord. Metformin was not measurable in any tissues of the embryo, including the gonads. The effect of metformin exposure on offspring was sex specific. The adult females that had been exposed to metformin *in utero* presented no clear reduction in fertility. However, the adult males that had been exposed to metformin during foetal life exhibited a 30% reduction in litter size compared with controls. The lower fertility was not due to a change in sperm production or the motility of sperm. Rather, the phenotype was due to lower sperm head quality – significantly increased spermatozoa head abnormality with greater DNA damage – and hypermethylation of the genomic DNA in the spermatozoa associated with lower expression of the ten-eleven translocation methylcytosine dioxygenase 1 (TET1) protein. In conclusion, while foetal metformin exposure did not dramatically alter gonad development, these results suggest that metabolic modification by metformin during the foetal period could change the expression of epigenetic regulators such as Tet1 and perturb the genomic DNA in germ cells, changes that might contribute to a reduced fertility.

## Introduction

Metformin is a biguanide molecule used as an oral insulin-sensitising drug for the treatment of metabolic disorders, such as type 2 diabetes, obesity, insulin resistance and polycystic ovary syndrome (PCOS). Metformin is used widely, with more than 150 million people in the world taking it each year (1). Metformin decreases hepatic glucose production and increases insulin sensitivity though inhibition of the respiratory complex I of mitochondria (2, 3). The reduction in mitochondrial activity leads to an increase in the intracellular adenosine monophosphate (AMP)/ATP ratio, which activates a protein kinase called AMP-activated protein kinase (AMPK) involved in the regulation of glucose homeostasis. Metformin is considered safe when administered either alone or in combination with other antidiabetic compounds such as insulin. The clinical advantages of metformin include normoglycaemia, low toxicity and no impact on weight gain or cardiotoxicity. Apart from its use in type 2 diabetes and obesity, its low toxicity has led it to be used increasingly to treat gestational diabetes and type 2 diabetes in pregnancy (4–7).

While metformin has not been approved for use during pregnancy (8), metformin administration during gestation is considered safe. Indeed, several retrospective and non-randomised studies have reported beneficial effects of metformin on pregnancy loss and pregnancy complications (9–13). However, the safety of metformin on the health of offspring is controversial (10, 14–16). For example, while a recent study showed that metformin administration to obese mothers during pregnancy prevents ovarian dysfunction in female offspring during adulthood (17), another report described increased obesity and higher body mass index (BMI) in offspring of metformin-treated mothers with PCOS (7).

Interestingly, in some cases of infertility associated with insulin resistance such as PCOS or to treat human gestational diabetes, a prescription for metformin could be given even in the first trimester of pregnancy (10, 11). Of note, toxicological studies have demonstrated that metformin is detectable in the human placenta and the concentration of metformin is similar in maternal blood and the foetal umbilical artery (18), leading to foetal exposure. Children exposed to metformin during gestation have higher fasting glucose levels and systolic blood pressure and increased sex hormone binding globulin (19, 20). In mouse models, metformin exposure causes long-term programming effects on the metabolic phenotype of offspring (15) associated with elevated adipose tissue.

Some reports have established that metformin could have both negative and positive consequences on parental fertility parameters depending on the dose, time, period of exposure, metabolic status and species (9, 21, 22). Positive effects have been described in adult men with metabolic syndrome, with an improvement in fertility through an increase in testosterone production (23, 24). In addition, metformin, alone or in combination with other drugs, has been shown to restore the ovarian function in women with PCOS and to improve foetal development, pregnancy outcomes and offspring health in gestational diabetes mellitus and type 2 diabetes mellitus cases (25). The foetal and prenatal periods are critical windows for development of the male and female reproductive tracts because they are sensitive to hormones and steroids (26, 27). Few studies have assessed the consequences of metformin exposure *in utero* on ovary or testis development in offspring and during adulthood. In mice, we reported that *in utero* metformin exposure during the first 15 days of pregnancy perturbed testis development, with a reduction in testis size, seminiferous tubule size and the number of Sertoli cells (28). In addition, *in vitro* analyses of direct metformin exposure to human and mouse foetal testis demonstrated a decrease in testosterone secretion (28). However, in humans there appears to be no effect of *in utero* metformin exposure on the testicular size of boys 3–7 years of age compared with *in utero* insulin exposure (29).

In a recent study, high fat diet–fed rat dams were treated with metformin from 1 week before mating until weaning; the adult female offspring of these dams showed improved ovarian function (17). In addition, clinical trials performed on the offspring of human mothers treated with metformin for PCOS during pregnancy showed that the offspring had an increase in obesity frequency and BMI (7, 30–34), indicating potential consequences on cardiometabolic health (7) in metformin-exposed children during adulthood. Taken together, these studies suggest that *in utero* metformin exposure could induce consequences on hormonal functions. Despite those studies, there is still little known regarding the influence of embryonic metformin exposure on the development of the male and female offspring’s reproductive system and fertility. In the present study, we investigated the consequences of *in utero* metformin exposure on the development of ovaries and testes of offspring during the neonatal period and their subsequent fertility in adulthood.

## Materials and Methods

### Animals

Twenty pregnant females (12 weeks-old) and 10 adult males C57BL/6 mice were obtained from Charles River (L’Arbesle, France), and about 40 F1 male and 28 F1 female issues of the breeding were housed under controlled photoperiods (light on from 08:00 h–20:00 h) with *ad libitum* access to food and water. All animal procedures were carried out in accordance with the European legislation for animal experimentation (Directive 86/609/EEC). The use of experimental animals in this study was approved by the ethics committee of Val de Loire (CEEA VdL, Comité d’Ethique pour l’Expérimentation Animal du Val de Loire, n°2012-12-11).

### Metformin Exposure

For *in vivo* exposure, metformin treatment started the morning a vaginal plug was detected, designated as embryonic day 0.5 of pregnancy (E0.5), until delivery. Twenty pregnant female mice were housed in individual standard cages during pregnancy and were treated with an average concentration of 250 mg/kg metformin in drinking water per day (Sigma, l’Isle d’Abeau Chesnes, France) (12 females with metformin and 8 with control water); the dose was adjusted according to the animal’s weight in the drinking water or only drinking water was used for control pregnant mice as described previously (35). Food and water consumption was measured every 48 h as described previously (36, 37). Immediately after birth, the metformin treatment was stopped and removed from the drinking water given to the dam and pups. Analyses of offspring were performed at 2 days postpartum (dpp) for ovarian analysis or at 5, 25 and 90 dpp for testicular analysis, representing newborn, childhood and adult stages of development, respectively. The experiments were performed in duplicate, with seven pregnant mice per condition. Adult males and females (90 dpp) that had been exposed *in utero* to metformin were subsequently crossed with three control males or females to assess fertility. The litter size was counted at birth.

All animals were weighed, anaesthetised by intraperitoneal injection of sodium pentobarbital and were euthanised by cervical dislocation. Visceral fat, testes, epididymides, seminal vesicles, ovaries, uteruses, placentas, embryos and biological fluids were collected. Some organs were weighed, fixed in 10% formalin and embedded in paraffin; others were frozen at -80°C.

### Glycaemia Analyses in Pregnant Mothers

Blood glucose was evaluated in female mice at E10 of pregnancy. They were fasted overnight before the oral glucose tolerance test (OGTT). In the morning, an intraperitoneal injection of a solution of 10% d-glucose at 2 g/kg was performed and at 0, 15, 30, 45, 60 and 90 min, post-injection the blood glucose level was measured in the tail vein with a ONE TOUCH^®^ Vita^®^ (LifeScan, Issy-les-Moulineaux, France). The experiment was performed on seven mice per condition (control *vs* metformin).

### Parameters of Fertility of Adult Offspring Exposed to Metformin (F1)

The anogenital distance (AGD) was measured using a calliper with a digital readout. The ovarian cycle was determined by vaginal smear examination performed in the morning (between 08:00 h and 10:00 h). Smears were stained with 0.5% methylene blue solution and examined under a light microscope. The oestrous cycle was classified into the following phases: The oestrous cycle was classified into the following phases: pro-oestrus, oestrus, and dioestrus, according to vaginal cell morphology. A normally cycling animal was defined as having 4–5-day interoestrous intervals.

The follicle number was quantified after staining an ovarian section with haematoxylin–eosin. Follicles were counted on every fifth section, using the oocyte nucleus as a marker, and the stage of follicular development was determined. Follicles were classified according to the shape and number of layers of somatic cells surrounding the oocyte: primordial (flattened cells) and primary (one layer of cuboidal cells). Oocytes were stained with a p63 antibody as described below. Atretic follicles were recognised based on the abnormal shape (clearly absent, shrinking or fragmented) of the oocyte or due to a large proportion of degenerating granulosa cells.

To assess the response of ovaries to gonadotropins, oocytes were retrieved following superovulation induction. Mice were injected intraperitoneally with 5 IU of equine chorionic gonadotropin (eCG), followed 46 h later with 5 IU of human chorionic gonadotropin (hCG, Intervet, Boxmeer, Holland). Twelve hours later, the oocytes were retrieved and counted and blood, tissues or embryos (at 5 days post coitum [dpc]) were immediately recovered.

To evaluate the morphometry of the testes, fixed testes were serially sectioned at a thickness of 7 μm. Round transverse sections of seminiferous tubule diameters were measured for each testis (n = 20 measurements per animal).

To evaluate sperm production, the epididymis was cut and suspended in 1 ml of 0.15 M NaCl buffer with 0.05% Triton X-100. Samples were homogenised and sonicated for 30 s. Sperm heads were counted with a haemocytometer, and after correction for sample volume and tissue weight, the concentration was determined.

To evaluate sperm morphology and motility, spermatozoa were retrieved from the cauda epididymis and incubated in M2 medium (Sigma) supplemented with 1% bovine serum albumin (BSA). At least 120 spermatozoa per animal were analysed and classified as normal or atypical (microcephalic, thin, deformed and curved head). The sperm suspension was also diluted for quantitative assessment of motility using a Hamilton–Thorne motility analyser (Hamilton-Thorne Biosciences, Beverly, MA) as described previously (38).

The parameters measured were: the percentage of motile sperm, the percentage of progressively motile spermatozoa, path velocity (VAP, average velocity/smoothed average position of the spermatozoa), progressive velocity (VSL, straight line distance between the beginning and the end of the track) and curvilinear line velocity (VCL, average velocity measured over the actual point-to-point track followed by the cell). For each mouse (n = 6), 1 000 spermatozoa were analysed at 37°C in 100-μm standard counting chambers (Leja, IMV Technologies, Aigle, France). All results are presented in **Table 1**.

**Table 1:** Weight of reproductive organs in adult males (90dpp) exposed *in utero* (mg of tissue)/body weight (g).

Age	25dpp		90dpp	
Treatment	Control	Metformin	Control	Metformin
**Testis**	2.30 ± 0.12	2.28 ± 0.05^ns^	3.71 ± 0.16	3.47 ± 0.18^ns^
	n=12	n=8	n=13	n=8
**Epididymis**	1.49 ± 0.19	1.28 ± 0.08^ns^	2.48 ± 0.10	3.78 ± 0.60^ns^
	n=8	n=8	n=4	n=7
**Seminal vesicles**	0.40 ± 0.14	0.16 ± 0.03^ns^	7.29 ± 0.67	6.96 ± 0.65^ns^
	n=10	n=8	n=5	n=8

Organ weights (mg) are normalized by body weight and noted as (mg of tissue per gram of body weight) as mean ± SEM. ns, not significant.

### Mass Spectrometry

Metformin was detected by MS analysis of the animal fluids and tissues in adult or in foetus (15 dpc) (39). The fluids were extracted using a protocol adapted from a previous study (40). Briefly, 10 μl of the liquid samples was combined with 30 μl of water, 20 μl of MeOH and 90 μl of MeCN. The solution was vortexed for 1 min, left to stand for 15 min and centrifuged at 3 000 g for 5 min. The supernatant was collected and dried using a Speedvac, then reconstituted using 100 μl of a 50:50 mixture of MeCN/0.1% FA.

The extracts were analysed in infusion mode on an LTQ orbitrap XL mass spectrometer (Thermo Fisher Scientific, Bremen, Germany) by selected ion monitoring (SIM). The source voltage and capillary temperature were set to 5 kV and 275°C, respectively. Analysis was performed in positive mode, using a resolution of 30 000 at *m*/*z* 200. The FTMS SIM acquired gain control (AGC) target was set to 100 000 and the SIM maximum injection time was set to 50 ms. MS/MS scans were performed by collision-induced activation (CID) using the linear ion trap. The collision energy was set to 35%, and the isolation window at ± 1 ppm.

MS imaging of the tissue sections was realised using an UltraFlex II matrix-assisted laser desorption/ionisation (MALDI) time-of-flight (TOF) instrument (Bruker Daltonics, Bremen, Germany) equipped with a Smartbeam laser having a repetition rate up to 200 Hz and controlled by FlexControl 3. 0 (Build184) software (Bruker Daltonics, Bremen, Germany). Embryos, ovaries, testes or uteruses were dissected and snap frozen in liquid nitrogen and stored at -80°C until use. E15 embryos or tissues were embedded in 2% carboxymethylcellulose (CMC), while the neonatal tissues were cryosectioned without additional preparation. Tissue sections (12 μm thick) were obtained by using a cryostat (Leica Microsystems, Nanterre, France) and mounted on indium/tin oxide (ITO)-coated glass slides (Bruker Daltonics). Next, 2,5-dihydrobenzoic acid (DHB) prepared at 15 mg/ml in 70% MeOH/0.1% Trifluoracetic acid (TFA) was used as a matrix and applied onto the tissue sections using the SunCollect instrument (SunChrom Wissenschaftliche Geräte GmbH, Friedrichsdorf, Germany). Image acquisition was performed in positive reflector mode, scanning at the *m*/*z* range of 60–1 000 m/z. The raster size was set at 30 µm (1 dpp) and 50 μm (E15), and the spectra per pixel was the result of an accumulation of 500 laser shots (using a SmartBeam laser operating at a frequency of 200 Hz). Spectra were normalised against the total spectrum count.

### Immunoblotting

Adipose tissue was prepared with three repeated freeze/thaw cycles in a lysis buffer containing 10 mM Tris (pH 7.4), 150 mM NaCl, 1 mM ethylenediaminetetraacetic acid (EDTA), 1 mM egtazic acid (EGTA), 0.5% IGEPAL and protease and phosphatase inhibitors (Sigma) as described previously (38). Proteins were submitted to SDS–polyacrylamide gel electrophoresis under reducing conditions. After transfer, the membranes were incubated overnight at 4°C with antibodies against two adipokines secreted by the adipose tissue (adiponectin and visfatin; Cell Signaling, Beverly, MA, USA), an enzyme involved in fatty acid oxidation (carnitine palmitoyl transferase I [CPT1]; Santa Cruz Biotechnology, Santa Cruz, CA, USA), a fatty acid synthase (FAS; Santa Cruz Biotechnology, Santa Cruz, CA, USA), and vinculin (Sigma). All antibodies were used at a dilution of 1:1 000. Horseradish peroxidase (HRP)-linked sheep anti-mouse IgG or donkey anti-rabbit IgG (1:10 000; Amersham Biosciences, Orsay, France) were used as the secondary antibody. The signal was detected by enhanced chemiluminescence (Amersham Pharmacia) and quantified using image analysis software (ImageJ, v 1.48, National Institutes of Health, Bethesda, MD, USA). The results are expressed as the intensity signal in arbitrary units, after normalisation by an internal standard (vinculin).

### Immunostaining

Deparaffinised and rehydrated ovarian or testicular sections were washed in phosphate-buffered saline (PBS). The sections were then permeabilised in boiling citrate solution (Vector Laboratories, Inc., AbCys, Paris, France) and washed with PBS. Spermatozoa were permeabilised with 100% methanol at -20°C and then a classical immunofluorescence protocol was applied. Nonspecific binding sites were saturated by incubating sections in 2% BSA in PBS for 15 min. Primary antibodies against DDX4 (Abcam, Cambridge, UK); DNA/RNA damage, using an antibody that recognises 8-hydroxy-2’-deoxyguanosine, 8-hydroxyguanine and 8-hydroxyguanosine (markers of DNA damage; Millipore, St Quentin en Yvelines, France); p63, phospho-(Ser10)Histone H3, CPT1 and FAS (Santa Cruz Biotechnology, Santa Cruz, CA, USA); 5 methylcytosine and Tet1 (Sigma); phospho-Ser 36-Histone H2B (ECM Biosciences, Versailles, KY, USA) (41); and peanut agglutinin (PNA) were used. Each antibody was diluted 1:100 in 1% BSA in PBS overnight at 4°C. The sections were then washed and incubated with an anti-rabbit or an anti-mouse secondary antibody conjugated to Alexa Fluor^®^ 488 (1:500; Invitrogen, Cergy Pontoise, France) for 1 h at room temperature in the dark. For the p63 antibody, the sections were incubated with a ready-to-use labelled polymer-HRP anti-mouse antibody for 30 min (DAKO Cytomation Envision Plus HRP System, Dako, Ely, UK). To visualise proteins, the sections were incubated in a 3,3′-diaminobenzidine solution (DAB) peroxidase substrate solution (Invitrogen). After a final wash, the sections were mounted with Fluoroshield™ (Vector Laboratories, Inc., AbCys, Paris, France).

### Testosterone and Lipid Assays

The frozen testis was homogenised and sonicated in 1X PBS (Basic Ultraturax, IKA^®^, WERKE, Staufen, Germany). The protein concentration was measured using a NanoDrop ND 1000 spectrophotometer (Thermo Scientific) to normalise the assay. The testosterone concentrations were determined by radioimmunoassay (RIA). The sensitivity of the assay was 15 pg/sample and the intra-assay coefficient of variation was 5.3% (38). Phospholipid, triglyceride and cholesterol concentrations were measured by using commercially available spectrophotometric assays (Biolabo, Maizy, France). The results obtained from testis lysates for each assay were normalised with the protein concentration. At least three animals were analysed as detailed in the figure legend.

### Analysis of Genomic DNA Methylation

Quantification of 5-methylcytosine was performed on mouse spermatozoa genome samples. Genomic DNA was extracted from cells treated with lysis buffer (10 mM Tris pH 8.0, 0.1 mM EDTA, 150 mM NaCl, 1% SDS) and proteinase K (10 mg/ml) for 4h. (10 mM Tris pH 8.0, 0.1 mM EDTA, 150 mM NaCl, 1% SDS) with proteinase K (10 mg/ml) for 4 h. Genomic DNA was purified using the QuiAMP DNA mini Kit (Qiagen, Germany). Quantification of 5-methylcytosine was performed with an ELISA using 100 ng of genomic DNA (Enzo Life Sciences, Villeurbanne, France).

The global methylation level of each DNA sample was measured using Luminometric Methylation Assay (LUMA), a pyrosequencing-based method (42), in two independent experiments. First, 200 ng of genomic DNA were digested by EcoRI and by either HpaII or MspI. Enzymatic digestion of DNA was performed using excess of restriction enzymes and a long period of incubation (3 h) to guarantee the efficiency of the reaction. Digested DNA was then diluted in Pyromark Annealing Buffer (Qiagen, Germany) and then pyrosequenced on a PyroMark Q24 sequencer (Qiagen; product no. 9001514) using PyroMark Gold Q24 Reagents (Qiagen; product no. 970802). The isoschizomers HpaII and MspI target the same DNA CCGG sequence, but HpaII is methylation sensitive and does not cleave methylated sites, while MspI is methylation insensitive. Pyrosequencing is used to sequence the overhangs left by both enzymes. During pyrosequencing, the proportion of incorporated C and G nucleotides at 5’-CG overhangs is directly related to the number of digested sites in the sample. The nucleotide dispensation order is defined as A;C;T;C;G;A, where the adenosine incorporation reflects the EcoRI digestion efficiency and the C incorporation reflects both HpaII and MspI digestions. We first normalized the peak height of C incorporation by the peak height of A incorporation to normalize for digestion efficiencies. We then calculated the peak height ratio of simultaneous C incorporation in HpaII and MspI digests, which is therefore representative of the DNA methylation level in the DNA sample and is close to 1 when the sample is unmethylated.

### Statistical Analyses

The data are presented as the mean ± SEM. Normality of the data and homogeneity of variances were determined by the Shapiro-Wilk and Bartlett tests, respectively. For normally distributed data, an unpaired t-test was used to compare metformin-treated animals with controls. For non-normally distributed data, the Mann–Whitney U test was used to compare the groups. To analyse the body weight at different dpp in female and male mice exposed to metformin *in utero*, we used One Way ANOVA with repeated measured. Quantification of labelling represented in percentages was analysed by using Fisher’s exact test. Differences in global methylation level between the two groups of mice were assessed by an unpaired t-test. Statistical analysis was performed by using GraphPad Prism 6 (La Jolla, CA, USA). Statistical significance is denoted by asterisks: *p ≤ 0.05; **p < 0.01; *** p < 0.001.

## Results

### Detection of Metformin in Biological Fluids

**Figure 1:**
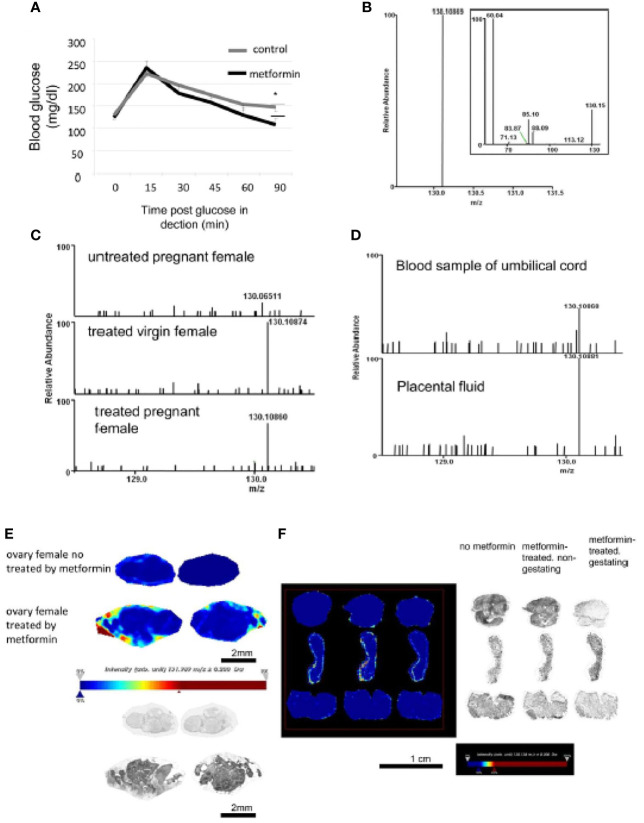
Detection of metformin in biological fluids of metformin-treated mothers by selected ion monitoring (SIM) mass spectrometry (MS). **(A)** An OGTT test was performed on E10 pregnant mice treated or not with metformin in drinking water. Values are expressed as mean ± SEM (ctrl n=6 vs met n=7). *p < 0.05. **(B)** High-resolution selected ion monitoring **(**SIM) spectrum of metformin standard, showing the [M+H]^+^ ion at m/z 130.1 (δmass = -0.26 ppm, theoretical mass 130.10972 computed from molecular formula C_4_H_12_N_5_). Inset shows the MS/MS spectrum of metformin. **(C)** SIM spectra of extracts from the blood samples of untreated pregnant female (top), metformin-exposed virgin female (middle, δmass = 0.14 ppm), and metformin-exposed pregnant female (bottom, δmass = 0.94 ppm). The m/z on all spectra are normalized relative to the intensity of the major peak in the scan range of m/z 128.5-131.5 (in this case, the [M+H]^+^ of metformin). **(D)** SIM spectra of extracts from blood recovered from umbilical cord (top, δmass = -0.32 ppm) and placental fluid (bottom, δmass = -0.68 ppm). **(E)** MS imaging of ovary from the mother mice. Image acquired at 30 μm spatial resolution. Pictures showed metformin signal (m/z 130.1) and intensity values are expressed in arbitrary units (a.u.) normalized against the total ion count with a low intensity in blue staining to high intensity in red staining as observed in the intensity bar below the picture. Picture with brightness are in the lower panel. Scale bar 2 mm. **(F)** MS imaging of uterus from mother mice non-exposed to metformin or non-pregnant or pregnant metformin-exposed female. Pictures showed metformin signal (m/z 130.1) and intensity values are expressed in arbitrary units (a.u.) normalized against the total ion count as observed in the intensity bar below the picture. Picture with brightness is in the right panel. Scale bar 1 cm. We can note an absence of metformin on both uterus samples.

**Figure 2:**
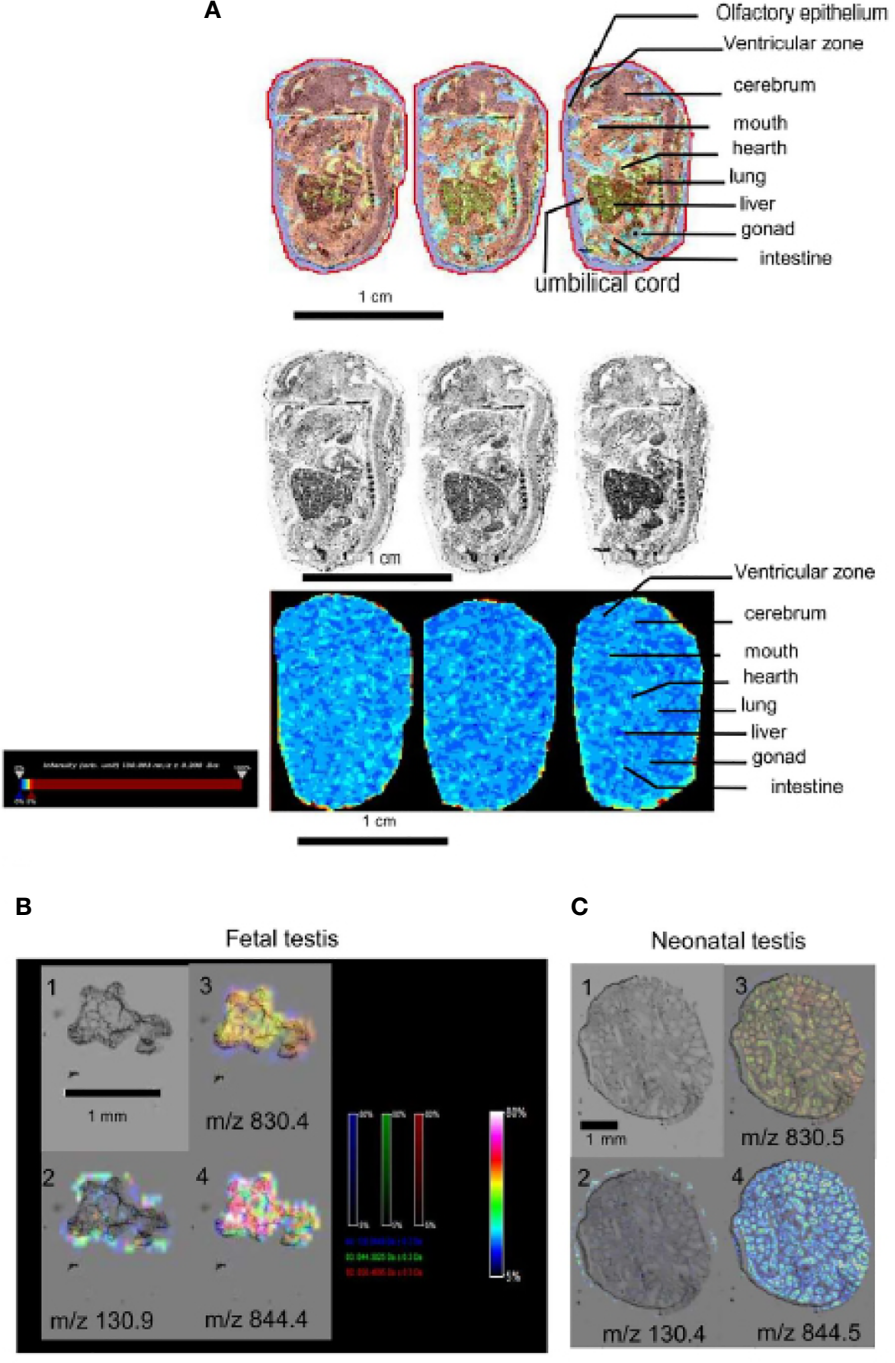
Detection of metformin in embryo by selected ion monitoring (SIM) mass spectrometry (MS). **(A)** Upper panel, picture with brightness and artificial color of sagittal section of a mouse embryo (15dpc) from a metformin-treated mother. Lower panel, MS imaging of metformin signal (m/z 130.1), images acquired at 30 μm spatial resolution. Intensity bar to the left of the picture. Scale bar 1 cm. **(B)** Embryonic testis (15dpc) and **(C)** neonatal testis (1dpp) exposed to metformin *in utero*. Panel 1: picture with brightness. Panel 2 pictures showing metformin signal (m/z 130.1). Panel 3 and 4 pictures showing positive signal (lipids) (m/z 830.4 and 844.4). Intensity values are expressed in arbitrary units (a.u.) normalized against the total ion count (low intensity: blue staining, high intensity: red staining), the intensity bar is in the middle of the image panel. We can note an absence of metformin on the testis. Scale bar 1 mm.

Metformin was administered to pregnant mice at E0.5. At E10, we confirmed the normoglycaemic properties of metformin of the mother by an OGTT (**Figure 1A**). The metformin standard was detected by MS/MS at 130.108 *m*/*z* (**Figure 1B**), and a peak was found in the blood of virgin and pregnant females mice exposed to metformin, but not in the control females (**Figure 1C**). Metformin was also detected in the umbilical cord and in amniotic fluid (**Figure 1D**). Metformin was localised directly in the external part of the ovary by MS (light blue, yellow to red staining) of females exposed to metformin (**Figure 1E**), but no cumulative metformin signal was observed in the uterus (**Figure 1F**). In addition, mass spectrometry imaging was not able to detect the metformin signal (130.1 *m*/*z*) in sections of foetal tissue (**Figure 2A**), including in the foetal testis at 15 dpc or at birth (**Figures 2B, C**). This was demonstrated by the absence of signal, in contrast to positive signals at 830.4 or at 844.5 *m*/*z* (**Figure 2B**).

### Effects of *In Utero* Metformin Exposure on Metabolic Parameters of Offspring

**Figure 3:**
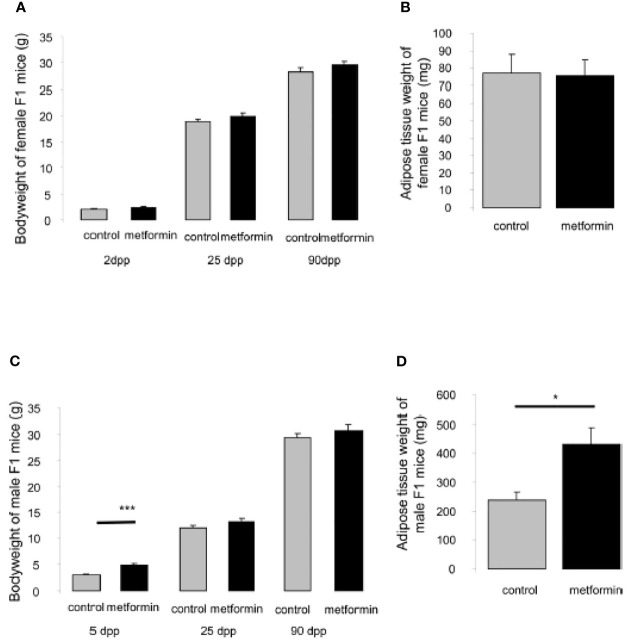
Body and adipose tissue weight of male and female offspring exposed *in utero*. **(A)** Body weight at 2dpp, 25dpp and 90dpp of female mice exposed to metformin *in utero*. **(B)** Weight of adipose tissue of adult female mice (90dpp) exposed to metformin *in utero*. **(C)** Body weight at 5dpp, 25dpp and 90dpp of male mice exposed to metformin *in utero*. **(D)** Weight of adipose tissue of adult male mice (90dpp) exposed to metformin *in utero*. Values are expressed as mean ± SEM (n=5-7). *p < 0.05, ***p < 0.001.

**Figure 4:**
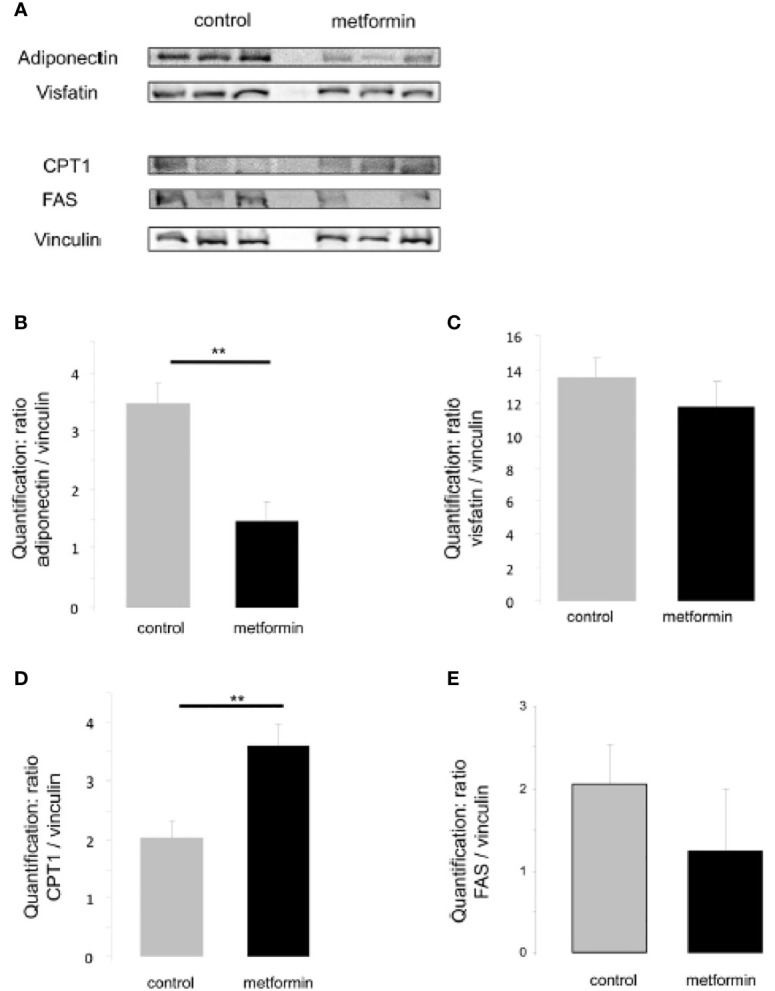
Expression of metabolic adipocyte markers in adult males exposed *in utero*. **(A)** Representative protein analysis (cropped blots) of adiponectin, visfatin, CPT1, FAS in adipose tissue extract from male mice that were exposed to metformin *in utero* **(B–E)** Quantification of proteins normalized against vinculin. (n=4-7). **p < 0.01.

At the neonatal stage (5 dpp), male but not female pups born from mice treated with metformin were heavier compared with those born to control mothers (**Figures 3A, C**); however, the difference in body weight was not maintained in adulthood (**Figures 3A, C**). In adulthood (90 dpp), we confirmed that females that had been exposed to metformin *in utero* had not developed an excess of adipose tissue, but males that had been exposed to metformin *in utero* had a 2-fold greater visceral fat weight compared with control males (**Figures 3B, D**). This elevated adipose tissue content was accompanied by a decrease in adiponectin protein expression (**Figures 4A, B**) but not for another adipokine hormone, visfatin (**Figures 4A, C**). The metabolic disorder in males that had been exposed to metformin *in utero* was confirmed by a dysregulation of CPT1 and FAS, markers of mitochondrial β-oxidation and fatty acid production, respectively. The increase in visceral fat weight was associated with an increase in CPT1 expression (**Figures 4A, D**) with no change in FAS expression (**Figures 4A, E**).

### Effects of *In Utero* Metformin Exposure on Gonadal Development

**Figure 5:**
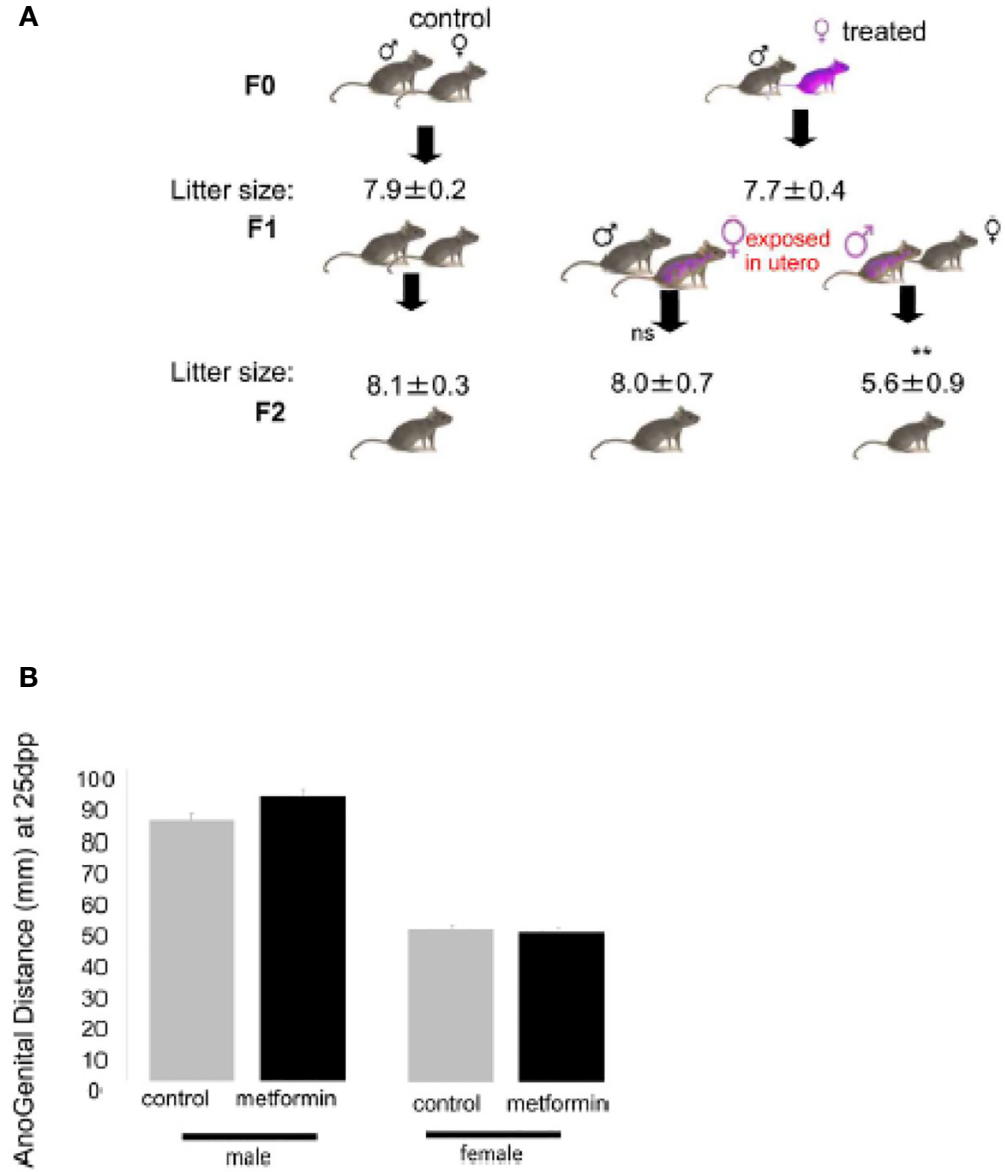
Effect on *in utero* metformin exposure on the fertility of adult male offspring. **(A)** Number of pups per litter from the F0 crossing and F1 crossing (n=8 litters per cross). Values of litter size are expressed as mean ± SEM. **(B)** Anogenital Distance (mm) of male and female mice exposed to metformin *in utero* at 25dpp.

To determine the consequence of metformin exposure during foetal life, both males and females were crossed with control mice and we observed a sex-specific effect on their fertility. Indeed, the females that had been exposed to metformin *in utero* presented the same litter size as control mice, while males that had been exposed to metformin *in utero* showed a 30% reduction in litter size compared with controls (**Figure 5A**). We noted that in both sexes the AGD was not affected by metformin, with the expected larger AGD in males and the shorter AGD in females (**Figure 5B**).

**Figure 6:**
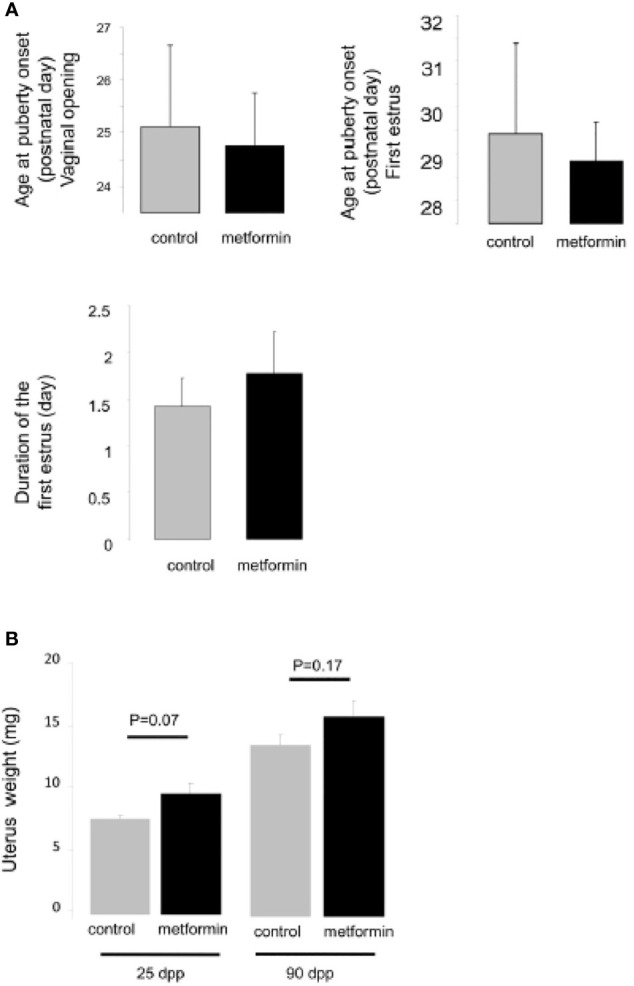
Age of puberty of female exposed in utero to metformin. **(A)** Age at vaginal opening (postnatal day), age at first ovulation (postnatal day), duration of the first estrus (day). **(B)** The weight of the uterus, a sensitive estrogenic tissue, from female mice exposed to metformin in utero at 25 dpp and 90 dpp.

**Figure 7:**
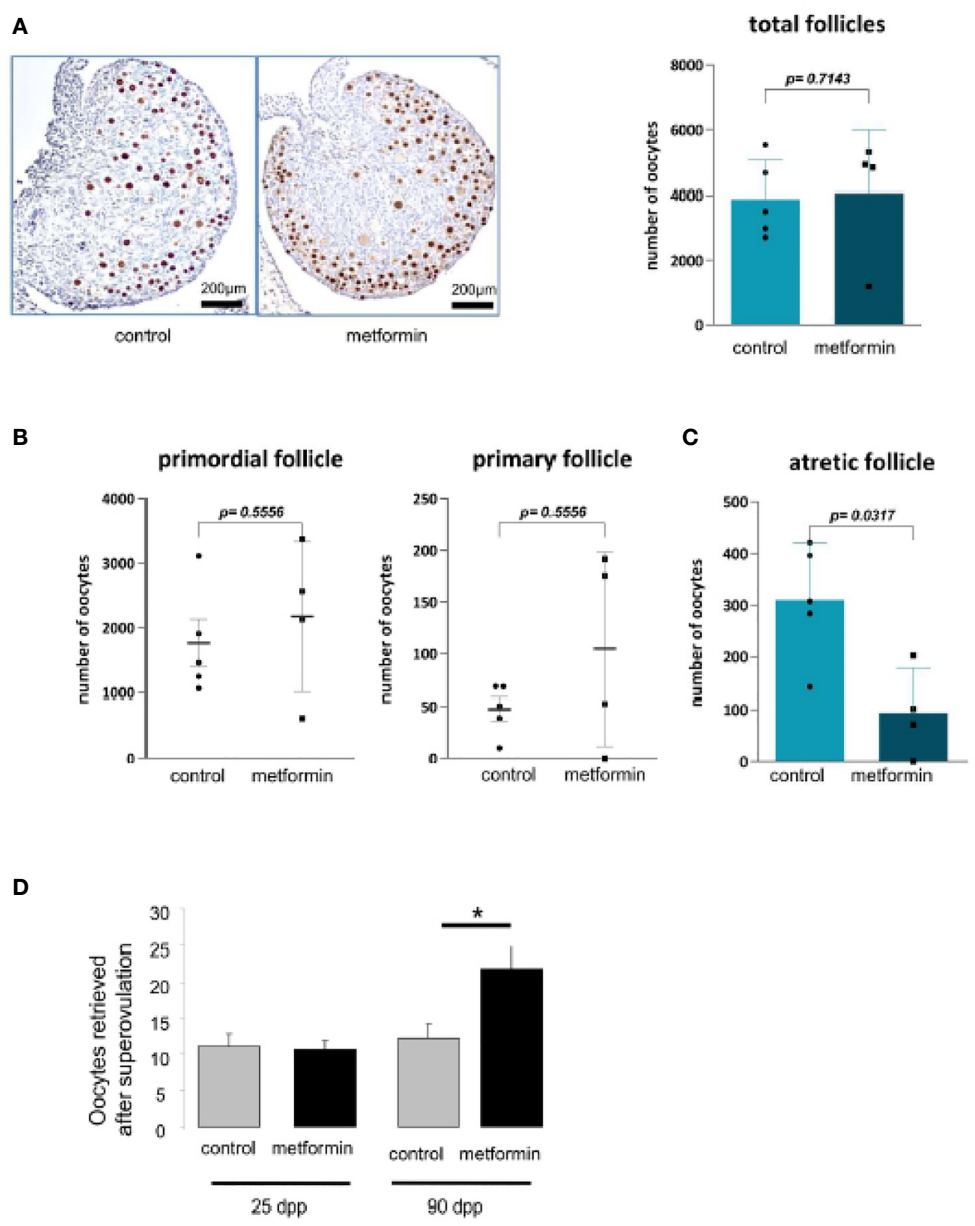
Morphology of ovary at birth. **(A)** Number of oocytes was measured on ovary from 2 dpp old female mice after a P63 immunostaining (n=5). **(B)** The quantification of follicle number was performed after a staining of ovarian section with haematoxylin–eosin. Follicles were classified according to the shape and number of layers of somatic cells surrounding the oocyte: primordial (flattened cells), and primary (one layer of cuboidal cells). **(C)** Quantification of atretic follicles **(D)** and number of oocytes retrieved after superovulation at 24 dpp and 90 dpp. Scale bar= 200 µm. White arrow indicates positive cell. *p < 0.05.

There was no effect on puberty onset in female offspring that had been exposed to metformin *in utero*: the age of the vaginal opening, the age of first oestrus and the duration of oestrus were similar to control female mice (**Figure 6A**). The uterus weight increased from childhood to adulthood but was the same between the groups (**Figure 6B**). We noted that the oocyte stock of primordial or primary follicles at birth was not altered by *in utero* metformin exposure (**Figures 7A, B**). However, we found fewer follicles had undergone atresia, suggesting more ‘viable and healthy’ follicles. Consistently, upon superovulation with exogenous gonadotropins, there was a 2 fold increase in oocytes of females that had been exposed to metformin *in utero* compared with control females (**Figures 7C, D**).

**Figure 8:**
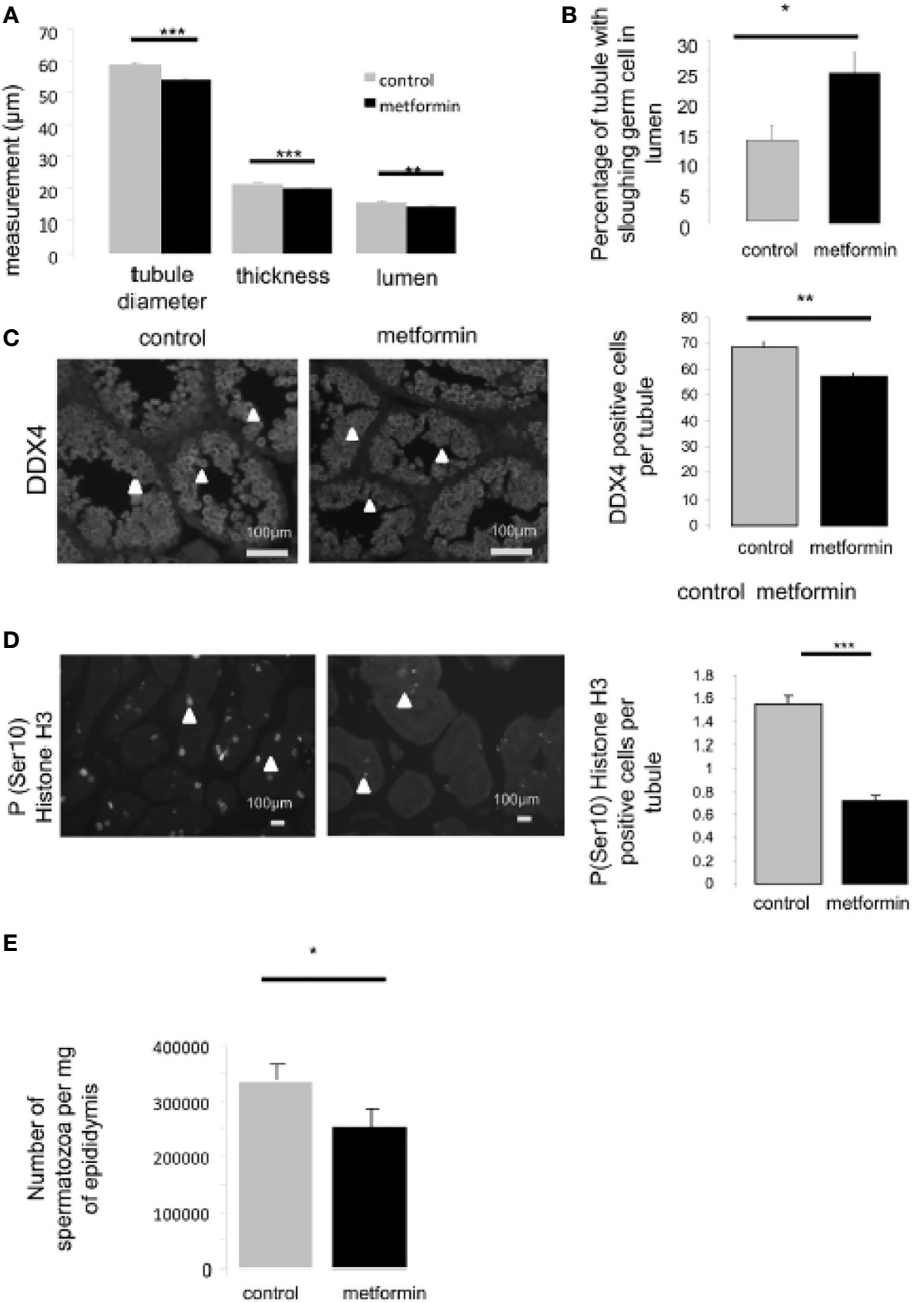
Testis morphology of 25dpp old male mice. **(A)** Seminiferous tubule diameter, thickness of seminiferous epithelium and lumen diameter were measured on 50 round seminiferous tubules (n=4). **(B)** Quantification of tubule with sloughing germ cells in lumen, (n=5, 150 seminiferous tubule per individual were counted). **(C)** Number of germ cells was quantified by immunohistochemistry against DDX4, Scale bar: 100 µm, **(D)** and against phospho-Ser10-Histone H3 (proliferative cells), a minimal of 100 round seminiferous tubules per mice was counted, (n=4). Quantification of positive cell per round seminiferous tubule is shown on the left side of the micrograph (n=4); there were counted on 150 seminiferous tubules per individual. Scale bar= 100µm. White arrow indicates positive cell. **(E)** Spermatic head concentration in epididymis, normalized against epididymis weight (control male n=4 vs male exposed *in utero* n=7). *p < 0.05, **p < 0.01, ***p < 0.001.

To determine the cause of the lower fertility in male offspring that had been exposed to metformin *in utero*, we collected morphometric measurements of the testes. At 25 dpp, the diameter of the seminiferous tubules was slightly but significantly reduced in males that had been exposed to metformin *in utero*, with a reduced thickness and lumen (**Figure 8A**). The tubules also presented a higher proportion of free sloughing germ cells in the lumen (**Figure 8B**). In the testes of males that had been exposed to metformin *in utero*, we noted a decrease in the number of germ cells per seminiferous tubule measured by DDX4 immunostaining compared with testes of control males (p < 0.01; **Figure 8C**); this change was associated with a reduction in the number of proliferative testicular cells immunostained with phospho-Histone H3 (Ser10) (**Figure 8D**). A consequence was fewer spermatozoa quantified in epididymides of males that had been exposed to metformin *in utero*.

Because testis development and spermatogenesis are controlled by androgens, we assessed the testosterone level. Systemic testosterone levels were similar between the two groups at 5, 25 and 90 dpp (**Supplemental Data 1A**). Consistently, the testis, epididymal and seminal vesicle weights, which are sensitive to androgen levels, were not different between the groups at 90 dpp (**Table 1**). Similarly to the adipose tissue, the metabolic markers CPT1 and FAS were significantly reduced in the testes of males that had been exposed to metformin *in utero* (**Supplemental data 1B**).

### Effects of *In Utero* Metformin Exposure on Sperm Parameters of Offspring

**Figure 9:**
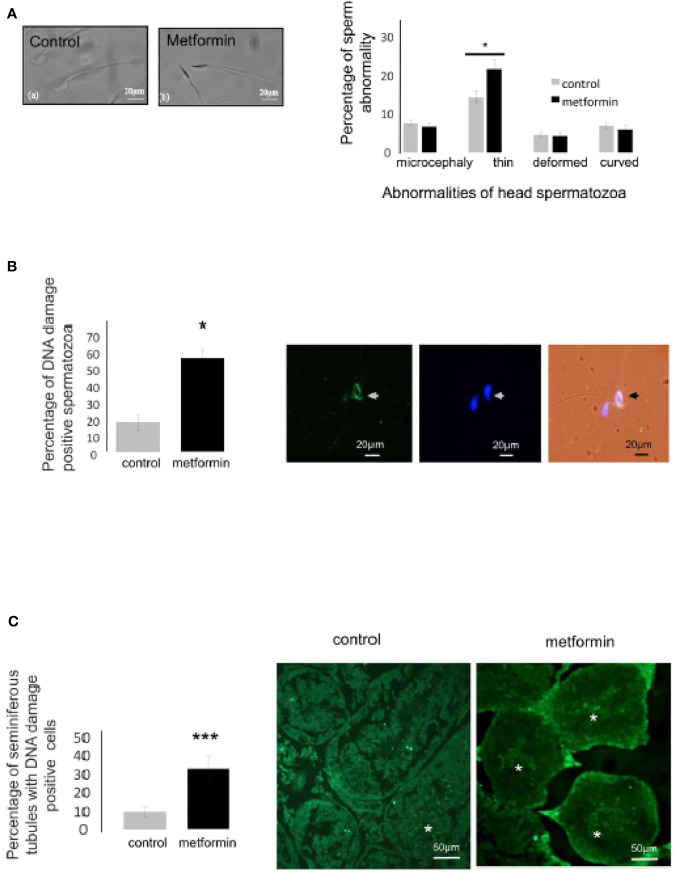
*In utero* metformin exposure induced a change in spermatozoa morphology. **(A)** Micrograph of a normal and thin head abnormality. Quantification of the head spermatozoa morphology normalized against the total number of spermatozoa counted (control,n=7; metformin, n=12), Scale bar: 20 µm. **(B, C)** Immunocytochemistry of spermatozoa and testis section against DNA damage (green), the nucleus was stained with DAPI (blue). Right panel, quantification of spermatozoa stained for DNA damage, Scale bar: 20 µm **(B)**, 50 µm **(C)** Values are expressed as mean ± SEM. *p < 0.05, ***p < 0.001.

Sperm production was not affected by *in utero* metformin exposure: analysis of sperm parameters by computer-assisted sperm analysis revealed no differences in the motility rate and velocity parameters between the groups (**Table 2**). Nonetheless, *in utero* metformin exposure significantly increased the incidence of sperm head abnormalities in adulthood (**Figure 9A**). Specifically, this exposure resulted in an increase in the proportion of sperm with a thin head and a 3-fold increase in spermatids and spermatozoa with DNA damage according to immunostaining (**Figures 9A–C**). The acrosome reaction occurred at the same rate in both groups (control: 56.90% ± 6.49% vs *in utero* metformin exposure: 51.22% ± 7.82%, p = 0.62; **Supplemental Data 1C**).

**Table 2:** Consequences of in utero metformin exposure on spermatozoa motility.

Spermatozoa parameters	Control (n=12)	Metformin (n=6)
**Static (%)**	40.61 ± 5.3	30.97 ± 3.7^ns^
**Progressive (%)**	16.94 ± 1.3	21.61 ± 2.6^ns^
**Motile (%)**	59.01 ± 5.6	69.00 ± 3.6^ns^
**VCL (µm/s)**	144.4 ± 7.5	145.0 ± 7.3^ns^
**VAP (µm/s)**	77.32 ± 3.9	76.02 ± 3.0^ns^
**VSL (µm/s)**	52.99 ± 3.0	52.07 ± 2.8^ns^

Values are expressed as mean ± SEM (control males n=12; males exposed in utero to metformin n=6). ns, not significant.

**Figure 10:**
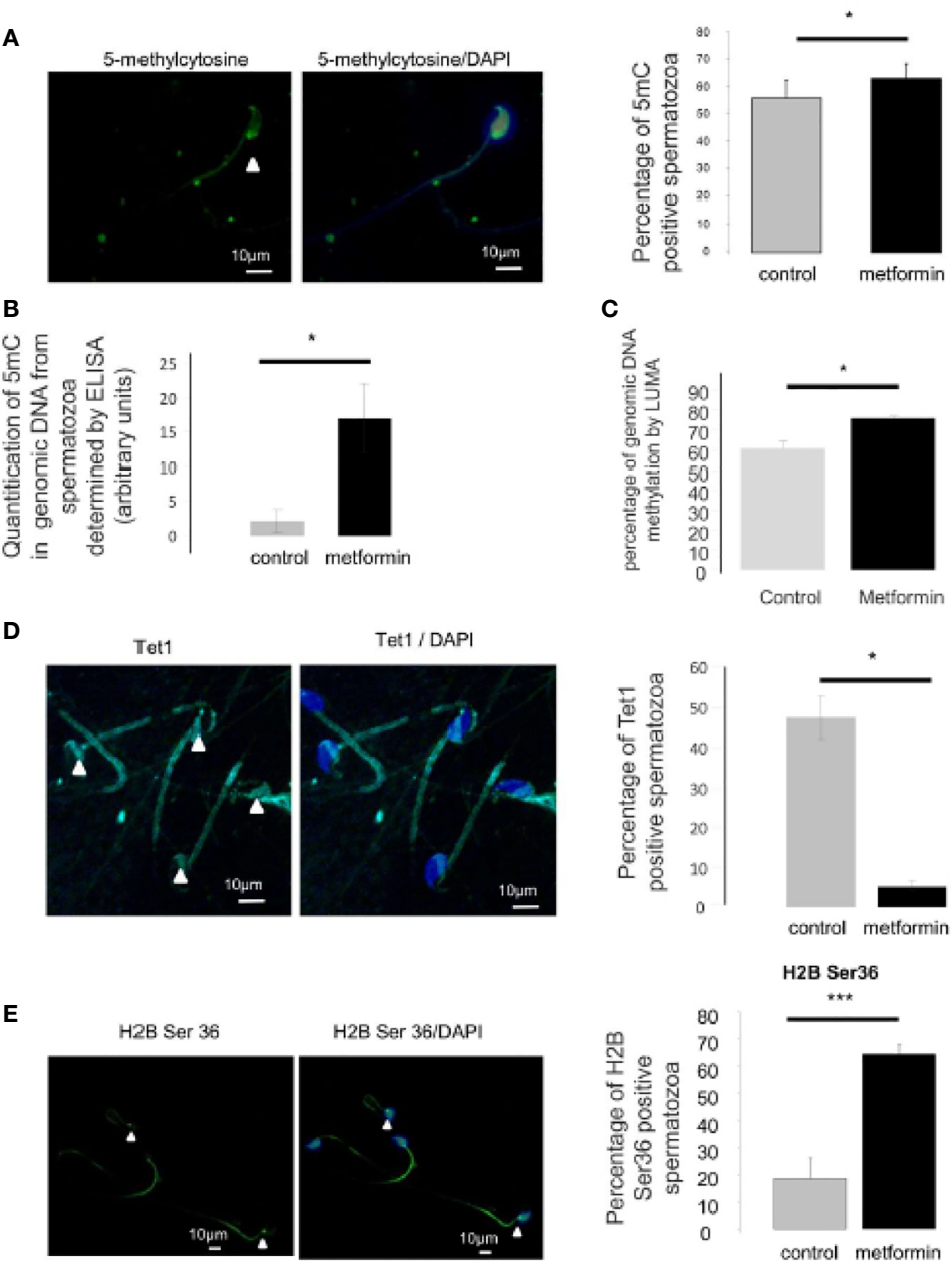
Analysis of epigenetic mark in spermatozoa. **(A)** Immunocytochemistry (green) against 5 methyl-cytosine in spermatozoa. Nucleus was stained with DAPI (blue). Quantification is shown on the right side of the micrographs (n=4 mice, at least 100 spermatozoa per individual were counted). Scale bar = 10 µm. **(B)** Percentage of 5mC in genomic DNA from spermatozoa was measured by ELISA assay and **(C)** percentage of genomic DNA methylation in epididymis cauda was analysed by LUMA. **(D)** Immunocytochemistry (green) against TET1 in spermatozoa. Nucleus was stained with DAPI (blue). Quantification is shown on the right side of the micrographs (n=4, at least 100 spermatozoa per individual were counted). Scale bar = 10 µm. **(E)** Immunocytochemistry (green) against phospho-Ser36-H2B in spermatozoa. Nucleus was stained with DAPI (blue). IgG was used as negative control. Quantification is shown on the right side of the micrographs (n=3-4, at least 100 spermatozoa per individual were counted). Scale bar = 10 µm. Values are expressed as mean ± SEM. *p < 0.05; ***p < 0.001.

Moreover, we evaluated the proportion of sperm with positive immunostaining for 5-methylcytosine and phosphorylation of the histone H2B at Ser36, which are markers associated with DNA and histone modification respectively. These markers have been detected in sperm cells (43–45) and are modulated by the metformin–AMPK pathway. Interestingly, the males that had been exposed to metformin *in utero* presented a slight, but significant, increase in the percentage of 5-methylcytosine-positive sperm cells, and a higher global methylation of sperm genomic DNA measured by ELISA and LUMA (**Figures 10A–C**). This change could be associated with a decrease in expression of the ten-eleven translocation methylcytosine dioxygenase 1 (TET1), an enzyme that catalyses the conversion of the modified DNA base 5-methylcytosine to 5-hydroxymethylcytosine (**Figure 10D**), in spermatozoa of mice that had been exposed to metformin *in utero* compared with control mice. In addition, *in utero* metformin exposure increased the intensity of the phosphorylated histone H2B (Ser36) by approximately 3 fold compared with control mice (p < 0.001; **Figure 10E**). The staining was localised at the lower region of the sperm head.

## Discussion

Our results demonstrate that *in utero* metformin exposure induced sex-specific metabolic and reproductive changes in adult offspring. These changes included a decrease in fertility only in males, manifested as a 30% decrease in litter size, more sperm abnormalities and DNA damage. It appears that the morphology of the testis was not affected by metformin because testis weight and testosterone production were unchanged. We observed metabolic changes outside the gonads, with an elevated level of visceral adipose tissue, as well as perturbations in FAS and CPT1 in the testis that could impact reproductive function.

Previous studies have shown that metformin administration around 250 mg/kg/day, as in the present study, is required to achieve a therapeutic effect in humans (36, 37, 46). In this study, we confirmed the activity of metformin during pregnancy by a quicker return to basal glucose levels during an OGTT. In addition, by using high-resolution MS, we detected metformin in the amniotic fluid and umbilical cord of the foetus, but there was no accumulation in foetal tissue. These results are similar to a study in humans – where the metformin concentration in the mother’s bloodstream (730 ± 440 ng/ml) was close to the concentration found in the umbilical cord (457 ± 335 ng/ml) (18) – and another mouse model, where the metformin level was similar in the mother and in foetal plasma (15, 47). Hence, metformin is transferred between maternal and offspring compartments. To our knowledge we have made the first attempt to measure metformin in male gonads using MS imaging. We did not detect metformin by MS imaging in the foetal testis, suggesting that while metformin could have activity in the foetus, we cannot speculate about its direct effects on embryonic gonads.

The effect of metformin on the metabolism of offspring has already been described in humans and rodents. We confirmed that only males that had been exposed to metformin *in utero* were heavier compared with control males and accumulated significantly more adipose tissue, suggesting changes in the body composition in those animals. The clear consequence of increased fat deposition in adulthood has already been described by Salomaki after prenatal exposure to metformin in a mouse model and in humans (15, 48). Prenatal metformin treatment resulted in a reduction in the expression of genes important for lipid, steroid and glucose metabolism (15, 48). Interestingly, because metformin has caloric restriction mimetic properties, the phenotype observed for *in utero* metformin treatment and for foetal caloric restriction are analogous. Thus, when maternal energy is restricted during the foetal period, more adipose tissue is deposited in male offspring compared with female offspring (49, 50). These studies present evidence that maternal diet during gestation affects the health and metabolic status of offspring later in life, perhaps through epigenetic regulation of imprinted genes involved in metabolism.

In both humans (51) and mouse models of obesity (52), the expression of the adipokine hormone adiponectin declines with obesity or increased BMI, and this reduction has direct consequences on the regulation of insulin sensitivity, satiety and inflammation (53). Our laboratory has shown that adiponectin and others adipokines could have modulatory effects on the gonads (54, 55). These findings suggest that dysregulation of the adiponectin pathway, as in our study, impacts the hypothalamic–pituitary–gonadal axis, sperm quality and, ultimately, fertility, as has been described previously (56, 57). It is also possible that the phenotype was accentuated because our mouse model did not present metabolic disturbances such as insulin resistance, which could be improved by metformin. Indeed, we only observed the phenotype of *in utero* metformin exposure in a healthy context, and we cannot discount that the effects of *in utero* metformin exposure on adults born to dams with metabolic disturbance could be different than what we have observed in this study.

In reproduction, several paper have cited an inhibitory effect of metformin on steroidogenesis in several species in ovarian and in testicular cells (28, 58, 59). During foetal testis development, two principal events occur: gametogenesis and initiation of steroidogenesis. A reduction in androgen production even at foetal stages can have significant consequences on gametogenesis and masculinisation of the male reproductive tract (60). In adult men with metabolic syndrome, unlike low testosterone production, after metformin administration, testosterone levels increased (23). In the present study, metformin exposure during the foetal period did not affect testosterone levels and thus there were no modifications of genitals, as attested by the AGD (61), as well as the testis, ovary and annexe gland morphology and weight. The absence of a metformin effect on sex differentiation has been reported recently (62)

Although in the females *in utero* metformin exposure did not alter metabolism or fertility, we noted that at birth the number of atretic follicles was low and could be associated with a higher number of oocytes retrieved following hyperstimulation. We can compare this result to those of Kong et al., showing, in rats, that females exposed *in utero* to 20 mg/kg/day of resveratrol (also an indirect activator of AMPK) present more follicles at 4 dpp and a reduction in the percentage of apoptotic oocytes (63). In db/db mice, metformin (0.1 mg/ml for 7 weeks) also increased the total number of oocytes compared with controls in the context of superovulation [pregnant mare serum gonadotropin (PMSG) and hCG] (64). Therefore, women exposed to prenatal undernutrition had a higher risk of early menopause (65, 66). This raises the question of a subtle deregulation of folliculogenesis or of sensitivity to gonadotropins as well as the risk of premature ovarian failure or of early menopause in females exposed to metformin during the foetal period.

A reduction in male fertility has been associated with altered quality of spermatozoa. We assessed whether sperm head abnormalities were associated with increased DNA damage and perturbations in the expression of chromatin modifications such as DNA methylation and histone phosphorylation (67). Modifications to histones and DNA in gametes and embryos are associated with reduced fertility (67, 68) and are a mechanism by which environmental factors may contribute to multi- or transgenerational disease (10, 69). In sperm recovered from adult males that had been exposed to metformin *in utero*, we observed a significant increase in DNA damage (about 3.6-fold more seminiferous tubules with germ cells with staining for DNA damage and 3.3-fold more spermatozoa with staining for DNA damage) associated with an increase in phosphorylated histone 2B (Ser36). AMPK-mediated increases in phosphorylated histone 2B (Ser36) have been observed in response to a broad range of metabolic stresses (70, 71). For example, histone H2B has been localised in the sperm head (43, 72) and in a study focusing on smokers who present a low sperm quality, the H2B/(H2B + Protamine1 + Protamine2) ratio were significantly higher in human sperm compared with non-smokers (73). In addition, we observed an increase in the intensity of 5-methylcytosine staining in offspring that had been exposed to metformin *in utero* and a strong difference in the 5-methylcytosine content in genomic DNA, confirming hypermethylation. Hypermethylated sperm have been shown to undergo apoptosis more readily, a phenomenon that reduces sperm viability (74, 75). Aberrations in DNA methylation in a genome-wide and in a single-gene manner have often been detected in spermatozoa of sub-fertile men (75–81). *In utero* undernutrition perturbs DNA methylation patterns in germ cells and mature sperm cells; these patterns are subsequently transmitted and maintained in somatic cells in such a way to increase disease risk in subsequent offspring (69). Metformin is considered a caloric restriction mimetic (82) and has been reported to change the methylome by targeting the major metabolic supply route of methyl groups that are required for DNA methylation (83, 84). The increase in the mitochondrial metabolite, 2- hydroxyglutarate, inhibits the enzyme involved in methylation (TET), provoking DNA hypermethylation (85). TET proteins are expressed in all germ cells and TET1 is detected in human spermatids (86). Interestingly, under our conditions, we observed a decrease in TET1 expression in spermatids and a concomitant increase in the methylation marker (5-methylcytosine) in spermatozoa from male offspring that had been exposed to metformin *in utero*. Therefore, *in utero* undernourishment caused by maternal nutrition modulates epigenetic marks that have consequences on lipid metabolism or appetite in later life and could also underlie transgenerational transmission through germline DNA methylation in sperm cells (87, 88). Taken together, we hypothesise that metabolic modification caused by *in utero* metformin exposure modifies the epigenetic programming of sperm cells. This hypothesis could be explored through an epigenomic sequence analysis evaluating transmission of DNA methylation patterns in sperm.

In conclusion, our results demonstrate that *in utero* metformin exposure induced subfertility and promoted metabolic perturbations in male offspring. Despite the observed effects on fertility and adiposity, our results were obtained in healthy (non-diabetic) mice. Nonetheless, in the context of gestational diabetes and PCOS, these observations raise questions about the link between *in utero* metformin exposure during foetal development and the long-term health and fertility of offspring in adulthood. Further research is needed in human subjects to elucidate the long-term effects of metformin on offspring health and fertility.

## Data Availability Statement

The original contributions presented in the study are included in the article/**Supplementary Material**. Further inquiries can be directed to the corresponding author.

## Ethics Statement

The animal study was reviewed and approved by CEEA VdL, Comité d’Ethique pour l’Expérimentation Animal du Val de Loire.

## Author Contributions

JQ, IF, and MS, mass spectrometry and tissue preparation. MF and PF, collected biological sample, samples preparation. MF, MB, RK, and PF, interpreted results and prepared manuscript. MF, CR, RK, and HA, Spermatozoa analysis. PF, MF, M-JG, and CC, immunohistochemistry and quantification. MF, CR, and JD, western blot and hormone assays. MF and PF, hormone assays and animal care, management, design protocol. All authors contributed to the article and approved the submitted version.

## Funding

This work was supported by the national program « FERTiNERGY » funded by the French National Research Agency (ANR). MF was supported by the Region Centre and Institut National de la Recherche Agronomique.

## Author Disclaimer

RK is identified as personnel of the International Agency for Research on Cancer/World Health Organization. The author alone is responsible for the views expressed in this article and she does not necessarily represent the decisions, policy or views of the International Agency for Research on Cancer/World Health Organization.

## Conflict of Interest

The authors declare that the research was conducted in the absence of any commercial or financial relationships that could be construed as a potential conflict of interest.

## Publisher’s Note

All claims expressed in this article are solely those of the authors and do not necessarily represent those of their affiliated organizations, or those of the publisher, the editors and the reviewers. Any product that may be evaluated in this article, or claim that may be made by its manufacturer, is not guaranteed or endorsed by the publisher.

## Supplementary Material

Supplementary Data Sheet 1
**(A)** Testosterone levels measured at 5 dpp, 25 dpp and 90 dpp. Values are expressed as mean ± SEM (n=5-8). **(B)** Immunohistochemistry against CPT1 and FAS in seminiferous tubules (n=4). Scale bar = 50 µm. Quantification of the intensity was shown on the right side of the micrograph (n=4). **(C)** Acrosome reaction occurred at the same rate in both groups. Scale bar = 10 µm.

## References

[1] He L Wondisford FE. Metformin Action: Concentrations Matter. Cell Metab (2015) 21(2):159–62. doi: 10.1016/j.cmet.2015.01.003 25651170

[2] El-Mir MY Nogueira V Fontaine E Avéret N Rigoulet M Leverve X. Dimethylbiguanide Inhibits Cell Respiration *via* an Indirect Effect Targeted on the Respiratory Chain Complex I. J Biol Chem (2000) 275(1):223–8. doi: 10.1074/jbc.275.1.223 10617608

[3] Leverve XM Guigas B Detaille D Batandier C Koceir EA Chauvin C. Mitochondrial Metabolism and Type-2 Diabetes: A Specific Target of Metformin. Diabetes Metab (2003) 29(4 Pt 2):6S88–94. doi: 10.1016/S1262-3636(03)72792-X 14502105

[4] Tang T Glanville J Orsi N Barth JH Balen AH. The Use of Metformin for Women With PCOS Undergoing IVF Treatment. Hum Reprod (2006) 21(6):1416–25. doi: 10.1093/humrep/del025 16501038

[5] Renato P. Metformin in Women With PCOS, Pros. Endocrine (2015) 48(2):422–6. doi: 10.1007/s12020-014-0311-1 24913417

[6] Foretz M Guigas B Bertrand L Pollak M Viollet B. Metformin: From Mechanisms of Action to Therapies. Cell Metab (2014) 20(6):953–66. doi: 10.1016/j.cmet.2014.09.018 25456737

[7] Hanem LGE Salvesen Ø Juliusson PB Carlsen SM Nossum MCF Vaage MØ. Intrauterine Metformin Exposure and Offspring Cardiometabolic Risk Factors (PedMet Study): A 5–10 Year Follow-Up of the PregMet Randomised Controlled Trial. Lancet Child Adolesc Health (2019) 3(3):166–74. doi: 10.1016/S2352-4642(18)30385-7 30704873

[8] Panchaud A Rousson V Vial T Bernard N Baud D Amar E. Pregnancy Outcomes in Women on Metformin for Diabetes or Other Indications Among Those Seeking Teratology Information Services. Br J Clin Pharmacol (2018) 84(3):568–78. doi: 10.1111/bcp.13481 PMC5809340 29215149

[9] Faure M Bertoldo MJ Khoueiry R Bongrani A Brion F Giulivi C. Metformin in Reproductive Biology. Front Endocrinol (2018) 9(November):1–12. doi: 10.3389/fendo.2018.00675 PMC6262031 30524372

[10] Bertoldo MJ Faure M Dupont J Froment P. Impact of Metformin on Reproductive Tissues: An Overview From Gametogenesis to Gestation. Ann Transl Med (2014) 2(6):55. doi: 10.3978/j.issn.2305-5839.2014.06.04 25333030 PMC4200661

[11] Ghazeeri GS Nassar AH Younes Z Awwad JT. Pregnancy Outcomes and the Effect of Metformin Treatment in Women With Polycystic Ovary Syndrome: An Overview. Acta Obstet Gynecol Scand (2012) 91(6):658–78. doi: 10.1111/j.1600-0412.2012.01385.x 22375613

[12] Glueck CJ Goldenberg N Pranikoff J Loftspring M Sieve L Wang P. Height, Weight, and Motor-Social Development During the First 18 Months of Life in 126 Infants Born to 109 Mothers With Polycystic Ovary Syndrome Who Conceived on and Continued Metformin Through Pregnancy. Hum Reprod (Oxford England) (2004) 19(6):1323–30. doi: 10.1093/humrep/deh263 15117896

[13] Glueck CJ Phillips H Cameron D Sieve-Smith L Wang P. Continuing Metformin Throughout Pregnancy in Women With Polycystic Ovary Syndrome Appears to Safely Reduce First-Trimester Spontaneous Abortion: A Pilot Study. Fertil Steril (2001) 75(1):46–52. doi: 10.1016/S0015-0282(00)01666-6 11163815

[14] Kovo M Weissman A Gur D Levran D Rotmensch S Glezerman M. Neonatal Outcome in Polycystic Ovarian Syndrome Patients Treated With Metformin During Pregnancy. J Maternal-Fetal Neonatal Med: Off J Eur Assoc Perinatal Med Fed Asia Oceania Perinatal Soc Int Soc Perinatal Obstet (2006) 19(7):415–9. doi: 10.1080/14767050600682370 16923696

[15] Salomäki H Vähätalo LH Laurila K Jäppinen NT Penttinen A-M Ailanen L. Prenatal Metformin Exposure in Mice Programs the Metabolic Phenotype of the Offspring During a High Fat Diet at Adulthood. PloS One (2013) 8(2):e56594. doi: 10.1371/annotation/abe54d92-1f87-4826-a0a5-ba55005f99b4 23457588 PMC3574083

[16] Vanky E Ødegård R. Metformin in Pregnancy – Safe or Sorry? Nat Rev Endocrinol (2018) 14(10):570–2. doi: 10.1038/s41574-018-0081-6 30120391

[17] Álvarez D Ceballo K Olguín S Martinez-Pinto J Maliqueo M Fernandois D. Prenatal Metformin Treatment Improves Ovarian Function in Offspring of Obese Rats. J Endocrinol (2018) 239(3):325–38. doi: 10.1530/JOE-18-0352 30334444

[18] Tertti K Laine K Ekblad U Rinne V Ronnemaa T. The Degree of Fetal Metformin Exposure Does Not Influence Fetal Outcome in Gestational Diabetes Mellitus. Acta Diabetol (2014) 51(5):731–8. doi: 10.1007/s00592-014-0570-6 24633859

[19] Carlsen SM Vanky E. Metformin Influence on Hormone Levels at Birth, in PCOS Mothers and Their Newborns. Hum Reprod (2010) 25(3):786–90. doi: 10.1093/humrep/dep444 20023292

[20] Rø TB Ludvigsen HV Carlsen SM Vanky E. Growth, Body Composition and Metabolic Profile of 8-Year-Old Children Exposed to Metformin In Utero. Scand J Clin Lab Invest (2012) 72(7):570–5. 10.3109/00365513.2012.712319 22935043

[21] Bertoldo MJ Guibert E Tartarin P Guillory V Froment P. Effect of Metformin on the Fertilizing Ability of Mouse Spermatozoa. Cryobiology (2014) 68(2):262–8. doi: 10.1016/j.cryobiol.2014.02.006 24556364

[22] Faure M Guibert E Alves S Pain B Ramé C Dupont J. The Insulin Sensitiser Metformin Regulates Chicken Sertoli and Germ Cell Populations. Reproduction (2016) 151(5):527–38. doi: 10.1530/REP-15-0565 26917452

[23] Casulari L Caldas A Domingues Casulari Motta L Lofrano-Porto A. Effects of Metformin and Short-Term Lifestyle Modification on the Improvement of Male Hypogonadism Associated With Metabolic Syndrome. Minerva Endocrinol (2010) 35(3):145–51. 20938417

[24] Morgante G Tosti C Orvieto R Musacchio MC Piomboni P De Leo V. Metformin Improves Semen Characteristics of Oligo-Terato-Asthenozoospermic Men With Metabolic Syndrome. Fertil Steril (2010) 95(6):2150–2. doi: 10.1016/j.fertnstert.2010.12.009 21194687

[25] Shpakov AO. Improvement Effect of Metformin on Female and Male Reproduction in Endocrine Pathologies and Its Mechanisms. Pharm (Basel) (2021) 14(1):42. doi: 10.3390/ph14010042 PMC7826885 33429918

[26] Svechnikov K Stukenborg J-B Savchuck I Söder O. Similar Causes of Various Reproductive Disorders in Early Life. Asian J Androl (2014) 16(1):50–9. doi: 10.4103/1008-682X.122199 PMC3901882 24369133

[27] O’Shaughnessy PJ Fowler PA. Endocrinology of the Mammalian Fetal Testis. Reproduction (2011) 141(1):37–46. doi: 10.1530/REP-10-0365 20956578

[28] Tartarin P Moison D Guibert E Dupont J Habert R Rouiller-Fabre V. Metformin Exposure Affects Human and Mouse Fetal Testicular Cells. Hum Reprod (Oxford England) (2012) 27(11):3304–14. doi: 10.1093/humrep/des264 22811314

[29] Tertti K Toppari J Virtanen HE Sadov S Rönnemaa T. Metformin Treatment Does Not Affect Testicular Size in Offspring Born to Mothers With Gestational Diabetes. Rev Diabetic Stud (2016) 13(1):59–65. doi: 10.1900/RDS.2016.13.59 26859658 PMC5291182

[30] Maple-Brown LJ Lindenmayer G Barzi F Whitbread C Connors C Moore E. Real-World Experience of Metformin Use in Pregnancy: Observational Data From the Northern Territory Diabetes in Pregnancy Clinical Register. J Diabetes (2019) 11(9):761–70. doi: 10.1111/1753-0407.12905 30680949

[31] Løvvik TS Carlsen SM Salvesen Ø Steffensen B Bixo M Gómez-Real F. Use of Metformin to Treat Pregnant Women With Polycystic Ovary Syndrome (PregMet2): A Randomised, Double-Blind, Placebo-Controlled Trial. Lancet Diabetes Endocrinol (2019) 8587(19):1–11. doi: 10.1016/S2213-8587(19)30002-6 30792154

[32] Nguyen L Chan SY Teo AKK. Metformin From Mother to Unborn Child – Are There Unwarranted Effects? EBioMedicine (2018) 35:394–404. doi: 10.1016/j.ebiom.2018.08.047 30166273 PMC6156706

[33] Tarry-Adkins JL Aiken CE Ozanne SE. Neonatal, Infant, and Childhood Growth Following Metformin Versus Insulin Treatment for Gestational Diabetes: A Systematic Review and Meta-Analysis. PloS Med (2019) 16(8):e1002848. doi: 10.1371/journal.pmed.1002848 31386659 PMC6684046

[34] Hanem LGE Stridsklev S Júlíusson PB Salvesen Ø Roelants M Carlsen SM. Metformin Use in PCOS Pregnancies Increases the Risk of Offspring Overweight at 4 Years of Age: Follow-Up of Two RCTs. J Clin Endocrinol Metab (2018) 103(4):1612–21. doi: 10.1210/jc.2017-02419 29490031

[35] Hou M Venier N Sugar L Musquera M Pollak M Kiss A. Protective Effect of Metformin in CD1 Mice Placed on a High Carbohydrate-High Fat Diet. Biochem Biophys Res Commun (2010) 397(3):537–42. doi: 10.1016/j.bbrc.2010.05.152 20573602

[36] Hou M Venier N Sugar L Musquera M Pollak M Kiss A. Biochemical and Biophysical Research Communications Protective Effect of Metformin in CD1 Mice Placed on a High Carbohydrate – High Fat Diet. Biochem Biophys Res Commun (2010) 397(3):537–42. doi: 10.1016/j.bbrc.2010.05.152 20573602

[37] Foretz M Hébrard S Leclerc J Zarrinpashneh E Soty M Mithieux G. Metformin Inhibits Hepatic Gluconeogenesis in Mice Independently of the LKB1/AMPK Pathway *via* a Decrease in Hepatic Energy State. J Clin Invest (2010) 120(7):2355–69. doi: 10.1172/JCI40671 PMC2898585 20577053

[38] Froment P Staub C Hembert S Pisselet C Magistrini M Delaleu B. Reproductive Abnormalities in Human Insulin-Like Growth Factor-Binding Protein-1 Transgenic Male Mice. Endocrinology (2004) 145(4):2080–91. doi: 10.1210/en.2003-0956 14726451

[39] Stübiger G Pittenauer E Allmaier G. MALDI Seamless Postsource Decay Fragment Ion Analysis of Sodiated and Lithiated Phospholipids. Anal Chem (2008) 80(5):1664–78. doi: 10.1021/ac7018766 18229894

[40] Sørensen LK Hasselstrøm JB. A Hydrophilic Interaction Liquid Chromatography Electrospray Tandem Mass Spectrometry Method for the Simultaneous Determination of γ-Hydroxybutyrate and Its Precursors in Forensic Whole Blood. Forensic Sci Int (2012) 222(1–3):352–9. doi: 10.1016/j.forsciint.2012.07.017 22917943

[41] Bertoldo MJ Guibert E Faure M Guillou F Ramé C Nadal-Desbarats L. Specific Deletion of AMP-Activated Protein Kinase (α1ampk) in Mouse Sertoli Cells Modifies Germ Cell Quality. Mol Cell Endocrinol (2016) 423:96–112. doi: 10.1016/j.mce.2016.01.001 26772142

[42] Karimi M Johansson S Ekström TJ. Using LUMA: A Luminometric-Based Assay for Global DNA-Methylation. Epigenetics (2006) 1(1):45–8. doi: 10.4161/epi.1.1.2587 17998810

[43] Champroux A Damon-Soubeyrand C Goubely C Bravard S Henry-Berger J Guiton R. Nuclear Integrity But Not Topology of Mouse Sperm Chromosome Is Affected by Oxidative DNA Damage. Genes (2018) 9(10):501. doi: 10.3390/genes9100501 PMC6210505 30336622

[44] Efimova OA Pendina AA Tikhonov AV Parfenyev SE Mekina ID Komarova EM. Genome-Wide 5-Hydroxymethylcytosine Patterns in Human Spermatogenesis are Associated With Semen Quality. Oncotarget (2017) 8(51):88294–307. doi: 10.18632/oncotarget.18331 PMC5687605 29179435

[45] Bridgeman SC Ellison GC Melton PE Newsholme P Mamotte CDS. Epigenetic Effects of Metformin: From Molecular Mechanisms to Clinical Implications. Diabetes Obes Metab (2018) 20(7):1553–62. doi: 10.1111/dom.13262 29457866

[46] Wilcock C Bailey CJ. Sites of Metformin-Stimulated Glucose Metabolism. Biochem Pharmacol (1990) 39(11):1831–4. doi: 10.1016/0006-2952(90)90136-9 2111705

[47] Gregg B Elghazi L Alejandro EU Smith MR Blandino-Rosano M El-Gabri D. Exposure of Mouse Embryonic Pancreas to Metformin Enhances the Number of Pancreatic Progenitors. Diabetologia (2014) 57(12):2566–75. doi: 10.1007/s00125-014-3379-5 PMC4417192 25249235

[48] Salomaki H Heinaniemi M Vahatalo LH Ailanen L Eerola K Ruohonen ST. Prenatal Metformin Exposure in a Maternal High Fat Diet Mouse Model Alters the Transcriptome and Modifies the Metabolic Responses of the Offspring. PloS One (2014) 9(12):1–22. doi: 10.1371/journal.pone.0115778 PMC4277397 25541979

[49] Léonhardt M Lesage J Croix D Dutriez-Casteloot I Beauvillain JC Dupouy JP. Effects of Perinatal Maternal Food Restriction on Pituitary-Gonadal Axis and Plasma Leptin Level in Rat Pup at Birth and Weaning and on Timing of Puberty. Biol Reprod (2003) 68(2):390–400. doi: 10.1095/biolreprod.102.003269 12533401

[50] Painter RC Westendorp RGJ de Rooij SR Osmond C Barker DJP Roseboom TJ. Increased Reproductive Success of Women After Prenatal Undernutrition. Hum Reprod (2008) 23(11):2591–5. doi: 10.1093/humrep/den274 PMC2569844 18658159

[51] Kern PA Gregorio GB Di Lu T Rassouli N Ranganathan G. Adiponectin Expression From Human Adipose Tissue. Diabetes (2003) 52(17):1779–85. doi: 10.2337/diabetes.52.7.1779 12829646

[52] Landrier JF Kasiri E Karkeni E Mihaly O Beke G Weiss K. Reduced Adiponectin Expression After High-Fat Diet Is Associated With Selective Up-Regulation of ALDH1A1 and Further Retinoic Acid Receptor Signaling in Adipose Tissue. FASEB J (2017) 31(1):203–11. doi: 10.1096/fj.201600263rr PMC5161515 27729412

[53] Lu M Tang Q Olefsky JM Mellon PL Webster NJG. Adiponectin Activates Adenosine Monophosphate-Activated Protein Kinase and Decreases Luteinizing Hormone Secretion in LbetaT2 Gonadotropes. Mol Endocrinol (2008) 22(3):760–71. doi: 10.1210/me.2007-0330 PMC2262174 18006641

[54] Estienne A Bongrani A Reverchon M Ramé C Ducluzeau P-H Froment P. Involvement of Novel Adipokines, Chemerin, Visfatin, Resistin and Apelin in Reproductive Functions in Normal and Pathological Conditions in Humans and Animal Models. Int J Mol Sci (2019) 20(18):4431. doi: 10.3390/ijms20184431 PMC6769682 31505789

[55] Bongrani A Elfassy Y Brun JS Ramé C Mellouk N Fellahi S. Expression of Adipokines in Seminal Fluid of Men of Normal Weight. Asian J Androl (2019) 21(5):528–30. doi: 10.4103/aja.aja_25_19 PMC6732888 31115360

[56] Dobrzyn K Smolinska N Kiezun M Szeszko K Rytelewska E Kisielewska K. Adiponectin: A New Regulator of Female Reproductive System. Int J Endocrinol (2018) 2018:1–12. doi: 10.1155/2018/7965071 PMC5949163 29853884

[57] Elfassy Y Bastard JP McAvoy C Fellahi S Dupont J Levy R. Adipokines in Semen: Physiopathology and Effects on Spermatozoas. Int J Endocrinol (2018) 2018:3906490. doi: 10.1155/2018/3906490 29971101 PMC6008818

[58] Yang PK Hsu CY Chen MJ Lai MY Li ZR Chen CH. The Efficacy of 24-Month Metformin for Improving Menses, Hormones, and Metabolic Profiles in Polycystic Ovary Syndrome. J Clin Endocrinol Metab (2018) 103(3):890–9. doi: 10.1210/jc.2017-01739 29325133

[59] Velazquez EM Sosa F Glueck CJ. Metformin Therapy in Polycystic Ovary Syndrome Reduces Hyperinsulinemia, Insulin Resistance, Hyperandrogenemia, and Systolic Blood Pressure, While Facilitating Normal Menses and Pregnancy. Metabolism (1994) 43(5):647–54. doi: 10.1016/0026-0495(94)90209-7 8177055

[60] Smith LB Walker WH. Hormone Signaling in the Testis. 4th ed. In: Knobil and Neill's Physiology of Reproduction: Two-Volume Set. Vol. 1. Elsevier North-Holland, Inc.: Elsevier (2014). p. 637–90. doi: 10.1016/B978-0-12-397175-3.00016-8

[61] Rouiller-Fabre V Muczynski V Lambrot R Lécureuil C Coffigny H Pairault C. Ontogenesis of Testicular Function in Humans. Folia Histochem Cytobiol Pol Acad Sci Pol Histochem Cytochem Soc (2009) 47(5):S19–24. doi: 10.2478/v10042-009-0065-4 20067889

[62] Forcato S Montagnini BG de Góes MLM da Silva Novi DRB Inhasz Kiss AC Ceravolo GS. Reproductive Evaluations in Female Rat Offspring Exposed to Metformin During Intrauterine and Intrauterine/Lactational Periods. Reprod Toxicol (2019) 87:1–7. doi: 10.1016/j.reprotox.2019.04.009 31055052

[63] Kong X-X Fu Y-C Xu J-J Zhuang X-L Chen Z-G Luo L-L. Resveratrol, an Effective Regulator of Ovarian Development and Oocyte Apoptosis. J Endocrinol Invest (2011) 34(11):e374–81. doi: 10.3275/7853. 21738004

[64] Sabatini ME Guo L Lynch MP Doyle JO Lee H Rueda BR. Metformin Therapy in a Hyperandrogenic Anovulatory Mutant Murine Model With Polycystic Ovarian Syndrome Characteristics Improves Oocyte Maturity During Superovulation. J Ovarian Res (2011) 4(1):8. doi: 10.1186/1757-2215-4-8 21605417 PMC3121715

[65] Khorram O Keen-Rinehart E Chuang T-D Ross MG Desai M. Maternal Undernutrition Induces Premature Reproductive Senescence in Adult Female Rat Offspring. Fertil Steril (2015) 103(1):291–8.e2. doi: 10.1016/j.fertnstert.2014.09.026 25439841 PMC4282592

[66] Wang N Huang Y Wen J Su Q Huang Y Cai L. Early Life Exposure to Famine and Reproductive Aging Among Chinese Women. Menopause (2019) 26(5):463–8. doi: 10.1097/GME.0000000000001259 30516712

[67] Carrell DT. Epigenetics of the Male Gamete. Fertil Steril (2012) 97(2):267–74. doi: 10.1016/j.fertnstert.2011.12.036 22289286

[68] Stuppia L Franzago M Ballerini P Gatta V Antonucci I. Epigenetics and Male Reproduction: The Consequences of Paternal Lifestyle on Fertility, Embryo Development, and Children Lifetime Health. Clin Epigenet (2015) 7(1):1–15. doi: 10.1186/s13148-015-0155-4 PMC4642754 26566402

[69] Martínez D Pentinat T Ribó S Daviaud C Bloks VW Cebrià J. In Utero Undernutrition in Male Mice Programs Liver Lipid Metabolism in the Second-Generation Offspring Involving Altered Lxra DNA Methylation. Cell Metab (2014) 19(6):941–51. doi: 10.1016/j.cmet.2014.03.026 24794974

[70] Bungard D Fuerth BJ Zeng P-Y Faubert B Maas NL Viollet B. ShelleyL.Berger. Signaling Kinase AMPK Activates Stress-Promoted Transcription *via* Histone H2B Phosphorylation. Science (2010) 329:1201–5. doi: 10.1126/science.1191241 PMC3922052 20647423

[71] Luense LJ Wang X Schon SB Weller AH Lin Shiao E Bryant JM. Comprehensive Analysis of Histone Post-Translational Modifications in Mouse and Human Male Germ Cells. Epigenet Chromatin (2016) 9(1):1–15. doi: 10.1186/s13072-016-0072-6 PMC4915177 27330565

[72] Shinagawa T Huynh LM Takagi T Tsukamoto D Tomaru C Kwak H-G. Disruption of Th2a and Th2b Genes Causes Defects in Spermatogenesis. Development (2015) 142(7):1287–92. doi: 10.1242/dev.121830 25742800

[73] Hamad MF Shelko N Kartarius S Montenarh M Hammadeh ME. Impact of Cigarette Smoking on Histone (H2B) to Protamine Ratio in Human Spermatozoa and its Relation to Sperm Parameters. Andrology (2014) 2(5):666–77. doi: 10.1111/j.2047-2927.2014.00245.x 25044670

[74] Barzideh J Scott RJ Aitken RJ. Analysis of the Global Methylation Status of Human Spermatozoa and its Association With the Tendency of These Cells to Enter Apoptosis. Andrologia (2012) 45(6):424–9. doi: 10.1111/and.12033 23121197

[75] Urdinguio RG Bayón GF Dmitrijeva M Toraño EG Bravo C Fraga MF. Aberrant DNA Methylation Patterns of Spermatozoa in Men With Unexplained Infertility. Hum Reprod (2015) 30(5):1014–28. doi: 10.1093/humrep/dev053 25753583

[76] Houshdaran S Cortessis VK Siegmund K Yang A Laird PW Sokol RZ. Widespread Epigenetic Abnormalities Suggest a Broad DNA Methylation Erasure Defect in Abnormal Human Sperm. PloS One (2007) 2(12):e1289. doi: 10.1371/journal.pone.0001289 18074014 PMC2100168

[77] Marques CJ Costa P Vaz B Carvalho F Fernandes S Barros A. Abnormal Methylation of Imprinted Genes in Human Sperm Is Associated With Oligozoospermia. Mol Hum Reprod (2008) 14(2):67–73. doi: 10.1093/molehr/gam093 18178607

[78] Khazamipour N Noruzinia M Fatehmanesh P Keyhanee M Pujol P. MTHFR Promoter Hypermethylation in Testicular Biopsies of Patients With non-Obstructive Azoospermia: The Role of Epigenetics in Male Infertility. Hum Reprod (2009) 24(9):2361–4. doi: 10.1093/humrep/dep194 19477879

[79] Hammoud AO Wilde N Gibson M Parks A Carrell DT Meikle AW. Male Obesity and Alteration in Sperm Parameters. Fertil Steril (2008) 90(6):2222–5. doi: 10.1016/j.fertnstert.2007.10.011 18178190

[80] Minor A Chow V Ma S. Aberrant DNA Methylation at Imprinted Genes in Testicular Sperm Retrieved From Men With Obstructive Azoospermia and Undergoing Vasectomy Reversal. Reproduction (2011) 141(6):749–57. doi: 10.1530/REP-11-0008 21389080

[81] Li L-H Donald JM Golub MS. Review on Testicular Development, Structure, Function, and Regulation in Common Marmoset. Birth Defects Res Part B Dev Reprod Toxicol (2005) 74(5):450–69. doi: 10.1002/bdrb.20057 16193499

[82] Dhahbi JM Mote PL Fahy GM Spindler SR. Identification of Potential Caloric Restriction Mimetics by Microarray Profiling. Physiol Genomics (2005) 23(3):343–50. doi: 10.1152/physiolgenomics.00069.2005 16189280

[83] Cuyàs E Fernández-Arroyo S Verdura S García RÁF Stursa J Werner L. Metformin Regulates Global DNA Methylation *via* Mitochondrial One-Carbon Metabolism. Oncogene (2017) 37(7):963–70. doi: 10.1038/onc.2017.367 29059169

[84] Zhong T Men Y Lu L Geng T Zhou J Mitsuhashi A. Metformin Alters DNA Methylation Genome-Wide *via* the H19/SAHH Axis. Oncogene (2017) 36(17):2345–54. doi: 10.1038/onc.2016.391 PMC5415944 27775072

[85] Yang M Soga T Pollard PJ Yang M Soga T Pollard PJ. Oncometabolites: Linking Altered Metabolism With Cancer Find the Latest Version: Review Series Oncometabolites: Linking Altered Metabolism With Cancer. J Clin Investig (2013) 123(9):3652–8. doi: 10.1172/JCI67228 PMC3754247 23999438

[86] Ni K Dansranjavin T Rogenhofer N Oeztuerk N Deuker J Bergmann M. TET Enzymes are Successively Expressed During Human Spermatogenesis and Their Expression Level Is Pivotal for Male Fertility. Hum Reprod (2016) 31(7):1411–24. doi: 10.1093/humrep/dew096 27141042

[87] Lee H-S. Impact of Maternal Diet on the Epigenome During In Utero Life and the Developmental Programming of Diseases in Childhood and Adulthood. Nutrients (2015) 7(11):9492–507. doi: 10.3390/nu7115467 PMC4663595 26593940

[88] Radford EJ Ito M Shi H Corish JA Yamazawa K Isganaitis E. In Utero Effects. In Utero Undernourishment Perturbs the Adult Sperm Methylome and Intergenerational Metabolism. Science (2014) 345(6198):1255903. doi: 10.1126/science.1255903 25011554 PMC4404520

