# Supplementary figures and images for: In Utero Exposure to Metformin Reduces the Fertility of Male Offspring in Adulthood
Source: Front Endocrinol (Lausanne). 2021 Oct 18;12:750145. doi: 10.3389/fendo.2021.750145 (PMC8565088; doi:10.3389/fendo.2021.750145)

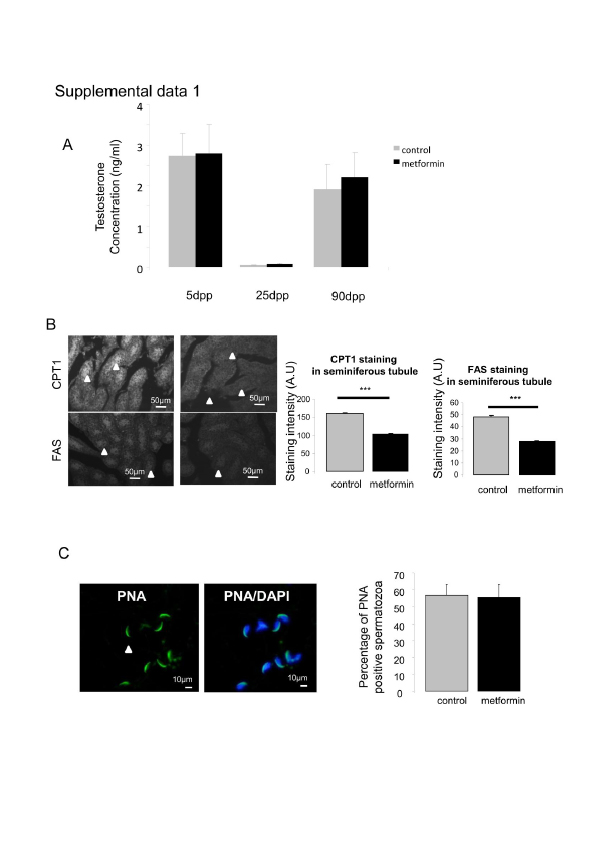

Supplement: Supplementary Data Sheet 1 — (A) Testosterone levels measured at 5 dpp, 25 dpp and 90 dpp. Values are expressed as mean ± SEM (n=5-8). (B) Immunohistochemistry against CPT1 and FAS in seminiferous tubules (n=4). Scale bar = 50 µm. Quantification of the intensity was shown on the right side of the micrograph (n=4). (C) Acrosome reaction occurred at the same rate in both groups. Scale bar = 10 µm. [file Image_1.jpeg]
